# Maternal Suicide Ideation and Behaviour During Pregnancy and the First Postpartum Year: A Systematic Review of Psychological and Psychosocial Risk Factors

**DOI:** 10.3389/fpsyt.2022.765118

**Published:** 2022-03-24

**Authors:** Holly E. Reid, Daniel Pratt, Dawn Edge, Anja Wittkowski

**Affiliations:** ^1^Division of Psychology and Mental Health, School of Health Sciences, Faculty of Biology, Medicine and Health, University of Manchester, Manchester, United Kingdom; ^2^Manchester Academic Health Science Centre, Manchester, United Kingdom; ^3^Greater Manchester Mental Health NHS Foundation Trust, Manchester, United Kingdom

**Keywords:** women, mothers, perinatal, suicide deaths, suicide attempts, suicidal ideation

## Abstract

Suicide is a leading cause of maternal death during pregnancy and up to a year after birth (perinatal period). Many psychological and psychosocial risk factors for maternal suicidal ideation and behaviour have been investigated. Despite this, there have been no attempts to systematically search the literature on these risk factors. Additionally, few studies have described how the risk factors for suicidal ideation, attempted suicides and suicide deaths differ, which is essential for the development of tools to detect and target suicidal ideation and behaviour. Seven databases were searched up to June 2021 for studies that investigated the association between suicidal ideation and/or suicidal behaviour and psychological/psychosocial risk factors in pregnant and postpartum women. The search identified 17,338 records, of which 59 were included. These 59 studies sampled a total of 49,929 participants and investigated 32 different risk factors. Associations between abuse, experienced recently or during childhood, and maternal suicide ideation, attempted suicide and death were consistently reported. Social support was found to be less associated with suicide ideation but more so with suicide attempts. Identifying women who have experienced domestic violence or childhood abuse and ensuring all women have adequate emotional and practical support during the perinatal period may help to reduce the likelihood of suicidal behaviour.

## Introduction

In the United Kingdom and Ireland, maternal suicide is the leading direct cause of death between 6 weeks and a year after the end of pregnancy and the second commonest direct cause of death occurring during or within 6 weeks of the end of pregnancy (1). This phenomenon is not exceptional to the United Kingdom; maternal suicide has been shown to be among the leading causes of death in other high-income countries, such as Australia (2), France (3), Italy (4), and the five Nordic countries (5). The United Kingdom and Ireland Confidential Enquiries into Maternal Deaths and Morbidity (1) reported that the rate of suicide during the perinatal period (i.e., pregnancy and the first postpartum year) has slightly increased over the last decade, with 0.60 deaths by suicide per 100,000 maternities reported in 2012–2014 and 0.63 deaths by suicide per 100,000 maternities reported in 2016–2018. Many more women who die by suicide will attempt suicide and a recent meta-analysis of 14 studies found the worldwide prevalence of suicide attempts during pregnancy was 680 per 100,000 and 210 per 100,000 during the postpartum period (6). Even greater numbers of women experience thoughts of suicide and/or self-harm, which is not only distressing, but may lead to a suicide attempt. The prevalence of self-harm ideation during the perinatal period was found to range from 5 to 14% (7).

Suicide prevention and risk reduction should be key intervention targets since maternal death by suicide has a profound effect on the woman’s child(ren), including loss of a primary care giver, feelings of separation and abandonment (8), increased rate of depressive symptoms (9), increased risk of bipolar disorder (10), and an increased risk of death by suicide themselves (11). Furthermore, maternal suicidal ideation alone has been associated with poorer child cognitive outcomes, including motor skills and language development (12).

Maternal suicides during the perinatal period appear to be distinguished from non-perinatal suicides in several ways. Firstly, these suicides tend to occur through more violent methods (e.g., hanging or jumping from a height) when compared with suicides of non-perinatal women (13–15). Secondly, during pregnancy and the first postpartum year most women have significantly more contact with healthcare professionals than at other times in their lives, but despite this, suicide has remained a leading cause of death in mothers. Thus, regular access to healthcare services alone may not be enough to encourage mothers to seek help for suicidal thoughts and/or behaviour and ultimately to avoid maternal death by suicide. An additional complexity is the supposed protective factor of being a parent; parenthood is associated with a lower risk of suicide in both men and women (16), but parenthood has also been found to be an impetus for suicide attempt among those who report high parenting stress (17). Given these unique characteristics of maternal suicidal ideation and behaviour, it is important to establish the risk factors associated with suicide during the perinatal period rather than extrapolate from research with non-perinatal samples.

Perinatal mental disorders are the most common complication of childbearing (18–20) and the early postpartum period is a particularly risky time for first and recurrent episodes of severe mental illness (21, 22), which may go some way towards explaining the high incidence of suicidal ideation and behaviour in the perinatal population. The risk of maternal suicide is significantly increased in mothers with first-onset severe psychiatric disorders compared to mothers with no psychiatric history (23). However, most people with a mental health problem never become suicidal and fewer than 5% of people admitted to hospital for the treatment of an affective disorder die by suicide (24). Therefore, the presence of a mental health problem has little predictive power and more specific risk factors for suicidal ideation, attempts and maternal death by suicide need to be identified.

In their epidemiological review of suicidal ideation during pregnancy only, Gelaye, Kajeepeta, and Williams (25) selected 57 studies for inclusion and identified intimate partner violence (IPV), less than 12 years of education and major depressive disorder as risk factors for antepartum suicidal ideation. In their review of 129 studies that investigated risk factors and clinical correlates of suicide during both pregnancy and postpartum, Orsolini et al. (26) identified that suicides were more likely to occur among younger women, during unwanted and unintended pregnancies and in those with psychiatric diagnoses. Despite the comprehensiveness of their review, Orsolini et al. (26) did not clarify whether the factors increased the risk of suicidal ideation or non-fatal suicidal behaviour or deaths, and only studies published in English were included. A more recent meta-analysis of 39 studies reported on the prevalence and correlates of self-harm regardless of suicidal intent, during the perinatal period (27). The review authors identified mental disorder, substance misuse, younger age, being unmarried, and obstetric and neonatal complications as key correlates of maternal self-harm. Taken together, these reviews highlight a very broad range of variables that have been investigated and they provide an indication of demographic groups of women more at risk of suicide during the perinatal period. However, most of these factors are “non-modifiable,” and therefore offer limited help when developing new interventions to reduce suicidal ideation and behaviour in perinatal women.

Previous studies have started to investigate a range of modifiable, psychological, and psychosocial factors that may increase a woman’s risk of suicide during the perinatal period, such as hopelessness and impact of childhood trauma (14, 28). A review of these psychological and psychosocial risk factors has yet to be conducted, but it would offer an important first step towards the development of new interventions targeting the reduction of suicidal ideation and behaviour. Therefore, this review aimed to (1) summarise the psychological and psychosocial risk factors associated with maternal suicide outcomes (i.e., suicidal ideation, suicide attempts, and suicide deaths) during the perinatal period; (2) describe how these risk factors differ between women experiencing suicidal ideation alone, women who attempt suicide, and women who die by suicide, during the perinatal period.

## Materials and Methods

The systematic review was reported in accordance with the Preferred Reporting Items for Systematic Reviews and Meta-Analyses (PRISMA) guidelines (29). The review protocol was registered with the International Prospective Register of Systematic Reviews (PROSPERO) on May 20, 2019 (registration number CRD42019107795).

### Search Strategy and Eligibility Criteria

A systematic search of the literature was conducted in seven databases: EMBASE, Medline, PsycINFO, CINAHL Plus, Maternity and Infant Care, Applied Social Sciences Index and Abstracts and Web of Science. A search was also conducted using Google Scholar. To uncover any relevant unpublished studies and grey literature, the Centre for Reviews and Dissemination databases, ProQuest Dissertations and Theses (United Kingdom and Ireland, Health and Medicine) and EThOS were also searched. The first author and university librarian developed a combined search strategy of free text terms and exploded Medical Subject Heading (MeSH) terms for the topics of suicide and the perinatal period, and MeSH terms were adapted for each database. The search strategy for MEDLINE is presented in Appendix A as an example. The reference lists of all papers identified for inclusion within this review and of existing reviews and position papers were also examined for any additional papers. The search was most recently conducted in June 2021.

The eligibility criteria were developed using the PICOS (Population, Intervention, Comparison, Outcome, Study design) framework (30) and are outlined in Table 1. Studies were included if the sample comprised pregnant and/or postpartum women aged 18 years old or over. Samples comprising adolescents only or majority adolescents were excluded because teenage motherhood brings its own unique challenges for a young woman, such as mothering while dealing with her own adolescent development, negative public attitudes and a lack of preparation for motherhood (31), which may confound the risk factors for suicide. Studies that only included women following a miscarriage, stillbirth, termination of pregnancy, or ectopic pregnancy were excluded because the factors associated with these losses are likely to be different from those experienced by suicidal women who have not experienced a loss or termination. Studies were required to measure at least one psychological or psychosocial risk factor. For the purposes of this review, definitions of psychological and psychosocial factors were adapted from O’Connor and Nock (32): Variables were deemed psychological factors if they represented cognitive factors (e.g., rumination, defeat, entrapment, agitation, belongingness, and burdensomeness) or personality and individual differences (e.g., hopelessness, impulsivity, perfectionism). Variables were deemed psychosocial factors if they represented social factors (e.g., exposure to death by suicide, social isolation) or negative life events (e.g., childhood adversities, traumatic life events during adulthood). Studies that measured psychological or psychosocial risk factors, which were found not to be correlated with a suicide variable (i.e., positively or negatively), were included to ensure the review was not biased towards statistically significant findings.

**TABLE 1:** Eligibility criteria for inclusion of studies in the review.

	Inclusion criteria	Exclusion criteria
**Population**	• Pregnant women and/or women during the first 12 months following birth (perinatal period). • From any location. • With or without a psychiatric diagnosis. • Women aged 18 years and over. For studies that included a mixed sample of adolescents and adults, the mean or median age of the sample needed to be 18 years or older. • Both inpatient and community samples.	• Women who had suffered a miscarriage or stillbirth, following a termination of pregnancy for any reason or ectopic pregnancies. • Adolescents (less than 18 years).
**Intervention (exposure)**	• Psychological/psychosocial risk factors. • Assessed using any objective or subjective measure.	• Investigate the presence of mental health problems (e.g., psychosis, major depressive disorder) but with no measurement of psychological and/or psychosocial factors.
**Comparison**	• Exposure versus non-exposure to the psychological/psychosocial risk factor(s) of interest.	• Purely descriptive studies (e.g., case report studies).
**Outcome**	• Suicide, which can include: – Suicide deaths – Suicide attempts – Suicidal ideation – Suicide planning – Self-harm ideation – Self-harm with suicidal intent or suicidal intent is unclear • Assessed using any objective or subjective measure.	• A focus on non-suicidal self-injury.
**Study design**	• Report original quantitative findings.	• Report qualitative findings only. • Reviews, practice recommendations or guidelines, comments, replies, letters, and opinion/position papers.

Given the wide-ranging definitions and measures of suicide outcomes as well as the difficulties of establishing suicidal intent, defining suicidal ideation and behaviour presents a challenge. For this review, initially, we included only studies that used any measure of suicide ideation, any measure of suicide attempts and suicide deaths. However, once we scrutinised the search results, we discovered that many studies that claimed to measure suicide ideation, used item 10 of the *Edinburgh Postnatal Depression Scale* (*EPDS*) (33) to assess ideation. The item asks the respondent whether “*the thought of harming myself has occurred to me*” and the respondent may answer “*yes quite often*,” “*sometimes*,” “*hardly ever*,” or “*never.*” There is uncertainty as to whether thoughts of harming oneself equate to suicidal ideation and the item does not explicitly ask the respondent whether the thoughts of self-harm are driven by suicidal intentions. However, endorsing “*yes quite often*” on the *EPDS* item 10 was found to be concordant with suicidality measured by the Clinical Interview Schedule–Revised measure of suicidality (34). In order to take an all-inclusive approach to defining suicide outcomes, we decided to include any studies that used the *EPDS* item 10, but we refer to the outcome measured by this item as “self-harm ideation” rather than suicide ideation. Studies that specifically focused on non-suicidal self-injury (i.e., self-harm with no suicidal intent) were excluded. To ensure all papers that met the eligibility criteria were identified, there were no restrictions on date of publication or language in which the study was reported.

### Study Selection, Data Extraction, and Analysis

**FIGURE 1:**
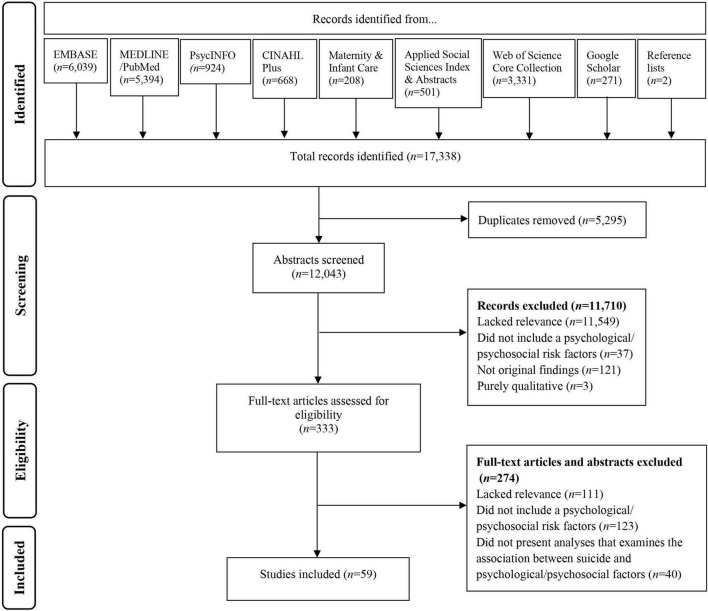
Flowchart of paper selection based on PRISMA guidance.

All database search results were imported into EndNote Online, a reference management service. The first author removed duplicates and screened all the identified studies to assess eligibility, according to the pre-specified inclusion and exclusion criteria. A peer outside of the research team also independently screened 50% of the identified abstracts, the percentage agreement between the independent raters was 99% (Cohen’s κ = 0.89). The first author and peer discussed and resolved any discrepancies regarding eligibility of studies. When a study was published in a language other than English, translation of the abstract was sought to identify whether the study met the inclusion criteria. If the study met the inclusion criteria, translation of the entire paper was sought. The number of studies identified, screened and selected are presented in the PRISMA flow diagram (see Figure 1). Data were extracted from the included studies into a table in Microsoft Word by the first author. To verify the accuracy of the data extraction, data from 50% of the included studies were also extracted independently by a peer outside of the research team, and any discrepancies or uncertainties were discussed and resolved.

Using the extracted data, a narrative synthesis was conducted following Popay et al.’s (35) guidance. Studies were organised into the four categories adapted from O’Connor and Nock (32), depending on the risk factors measured: negative life events, social factors, cognitive factors and personality and individual differences. This then allowed for clustering of studies depending on the suicide outcome investigated and the interrogation of similarities and differences between the clusters and the studies in each cluster.

### Risk of Bias and Quality Assessment

The studies included in the review were subject to a methodological quality assessment using the Quality Assessment Tool for Studies with Diverse Designs (QATSDD) (36). The review eligibility criteria are inclusive of a range of study designs and therefore the QATSDD was selected because it allows for the comprehensive examination of studies that employ different designs but address similar research questions. The QATSDD tool consists of a total of 16 criteria, two of which are solely applicable to quantitative studies and another two of the criteria are solely applicable to qualitative studies. Therefore, only mixed methods studies are assessed *via* all 16 criteria, and quantitative or qualitative studies are assessed by 14 criteria. Each criterion is weighted equally and rated 0 (not at all), 1 (very slightly), 2 (moderately), or 3 (complete) using the scoring guidance notes. As this review only included quantitative studies (no mixed methods studies were identified), a total score out of a possible 42 points was calculated for each study (i.e., a maximum score of 3 for 14 criteria). The total percentage was also calculated to provide an overall indication of methodological quality and similarly to the guide outlined by Gillham and Wittkowski (37), a percentage score over 75% was considered “high” quality, 50–75% “good,” 25–49% “moderate,” and below 25% “poor.” Furthermore, the percentage of studies that scored 1, 2, or 3 for each item was calculated to give an indication of how many of the studies addressed each of the 14 criteria. The first author rated all studies and made notes to elaborate on the QATSDD scoring guidelines to provide more detailed, tailored guidance to ensure consistency when scoring. A peer also rated 51% of the studies independently, using the additional scoring guidance notes provided by the first author. The percentage agreement between the independent raters was 86% before differences were discussed and resolved. None of the studies were excluded due to a “low” quality rating in order to ensure a broad range of risk factors were reviewed, although studies with particularly low ratings are highlighted and their methodologies discussed in the narrative synthesis.

## Results

### Study Characteristics

The systematic search strategy identified 17,338 titles (see Figure 1). After removal of duplicates and screening of abstracts, 333 full text publications were evaluated. Once publications that did not meet inclusion criteria and/or lacked relevant data were removed, 59 studies reporting quantitative associations between suicide outcomes and psychological and/or psychosocial factors during the perinatal period were included (see Supplementary Table 1 for study characteristics).

The 59 studies sampled a total of 49,929 participants and sample sizes ranged from 28 to 5,960. Twenty-three of the studies included pregnant women alone, 17 included postpartum women alone and 19 studies included both pregnant and postpartum women. Studies were conducted in a wide range of locations: 13 were conducted in the United States, 14 in Asia, 14 in Africa, nine in South America, seven in Europe, one in Australia, and one did not specify the location (38). Of the 59 studies included, 36 were cross-sectional in design (61%), 18 used a cohort design (31%), four were case-control studies (7%), and one used a mixed cohort and cross-sectional design whereby 121 of the 748 participants were seen at two time points (39).

Studies could be categorised depending on the suicide outcome measured: 43 studies measured suicidal or self-harm ideation alone (73%), 13 studies measured suicidal or self-harm ideation and suicide attempts (22%) either using a combined measure that assessed both the ideation and the attempts or *via* multiple measures that assessed the ideation and attempts separately, one study measured suicide attempts alone (2%) and two studies measured suicide deaths alone (3%).

**FIGURE 2:**
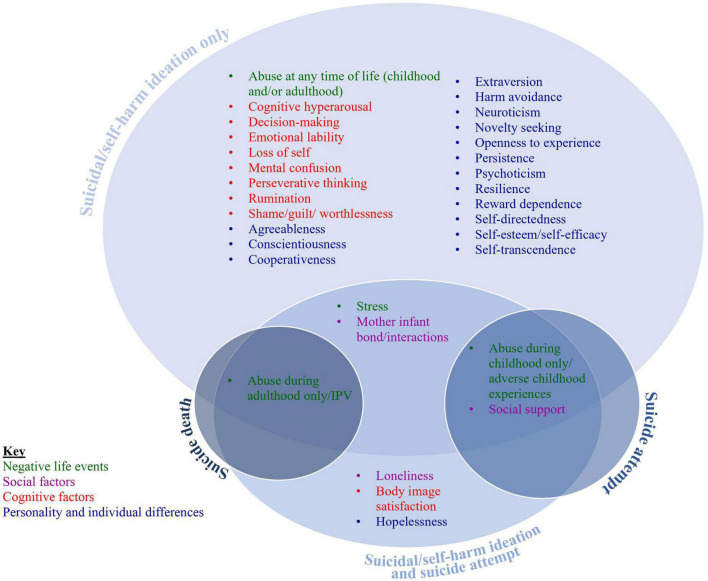
Diagrammatic illustration of the included risk factors and suicide outcomes.

A varied range of 32 psychological and psychosocial factors were investigated across the 59 studies. Those 32 studies were then grouped into the four categories adapted from O’Connor and Nock (32): negative life events, social factors, cognitive factors, and personality and individual differences. Figure 2 illustrates the risk factors and suicide outcomes measured.

### Risk of Bias and Quality Assessment of the Included Studies

Overall quality ratings for the 59 studies included in this review ranged between 33 and 86%, 44 of the studies were considered “good” quality (50–75%), 9 studies were “high” quality (>75%), and 6 were of “moderate” quality (25–49%). Ratings for each study on each of the QATSDD criteria are outlined in Supplementary Table 2. As no qualitative studies were included, criteria 11 and 14 were not utilised and removed. As evident in Supplementary Table 2, user involvement in the design of the studies was relatively poor (criterion 15), only seven studies used a pilot study, of which three used any pilot study feedback to inform changes to the study design and only one of the studies (40) consulted stakeholders about the study procedures. However, all 59 studies included some description of the research setting (criterion 3), recruited samples representative of perinatal women (criterion 5), used suitable methods of analysis (criterion 12), and included some explanation of choice of analytic method (criterion 13).

### Negative Life Events

Negative life events were commonly investigated as risk factors for perinatal suicide, with 47 studies investigating psychological/psychosocial risk factors that could be categorised as negative life events. The methodological quality ratings of these 47 studies ranged from 36 to 86% with 35 considered “good” quality, 7 were “high,” and 5 “low” quality. These negative life events could be clustered into four groups of very similar risk factors: experiences of abuse during childhood only or adverse childhood experiences, experiences of abuse at any time of life (childhood and/or adulthood), experiences of abuse during adulthood only or intimate partner violence (IPV), and stress (e.g., stress experienced during pregnancy or the postpartum, exposure to traumatic life events, post-traumatic stress symptoms, and 1st degree relative suicide).

#### Experiences of Abuse During Childhood Only or Adverse Childhood Experiences

Experiences of childhood abuse had significant associations with maternal suicide and self-harm ideation, and reports of physical childhood abuse appeared to be one of the strongest predictors across study designs. Sit et al.’s (41) cross-sectional findings from a sample of 628 postpartum women demonstrated that childhood physical abuse increased the odds of self-harm ideation by 68%, but childhood sexual abuse was not significantly associated with self-harm ideation. In their matched case-control study of 255 pregnant women, Leeners et al. (42) also showed childhood physical abuse increased the odds of suicide ideation by 20%; however, childhood sexual abuse was not significantly associated with suicide ideation, although there is limited information regarding whether the suicide ideation was experienced during the perinatal period or at another time during the participants’ lives. Moreover, in Giallo et al.’s (43) longitudinal study of 1,507 women, the univariate analysis revealed that experiences of childhood physical abuse increased the odds threefold and childhood sexual abuse increased the odds almost twofold of reporting self-harm ideation persistently at each time point (3 months, 6 months, 12 months, 18 months, and 4 years postpartum). However, in Giallo et al.’s (43) multivariable model, childhood sexual abuse was no longer a significant predictor of self-harm ideation.

It is not only abusive experiences during childhood that have been investigated. Doi and Fujiwara (44) assessed adverse childhood experiences, which included asking participants about parental death, parental divorce, parental mental illness, neglect, economic hardship, and IPV against their mother as well as about physical and psychological abuse. The authors found that postpartum women with three or more adverse childhood experiences were almost five times more likely to experience recent self-harm ideation.

Experiences of childhood trauma were also strongly associated with suicidal behaviour. In one of only three papers in this review to provide separate data for those reporting suicidal ideation only and suicide attempt, Levey et al. (45) discovered that a history of childhood abuse increased the odds of suicidal ideation 2.57-fold, increased the odds of suicide planning almost threefold and increased the odds of suicide attempt 2.43-fold in a sample of 2,062 pregnant women. In their longitudinal study that followed 306 women from pregnancy to 16 months postpartum, Martini et al. (46) identified that childhood abuse or rape was significantly associated with suicidality (defined as thoughts of death or self-harm, suicide planning or suicide attempt).

Gressier et al. (28) examined suicide attempts and the association with childhood abuse and adverse childhood experiences, using a database created by the French Network of Mother and Baby Units (MBUs) which contains information about perinatal women admitted to 16 MBUs (13 in France, 3 in Belgium) between 2001 and 2010. The authors retrospectively assessed 1,439 women for suicide attempts and of these, 105 had attempted suicide during the postpartum period, and 49 had made attempts during pregnancy. The study compared three groups: women who attempted suicide during pregnancy, women who attempted suicide during the postpartum period and women who did not attempt suicide. There was no difference across the groups on measures of foster care in childhood, maltreatment in childhood or childhood sexual abuse.

In summary, consistent evidence suggests that physical abuse experienced during childhood, rather than sexual abuse, increases the odds of perinatal suicide and self-harm ideation. The findings also suggest that childhood abuse increases the odds of suicide planning but there are mixed results regarding its association with suicide attempts during the perinatal period.

#### Experiences of Abuse at Any Time of Life

One study could not be included in Sections “Experiences of Abuse During Childhood Only or Adverse Childhood Experiences” or “Experiences of Abuse During Adulthood Only or Intimate Partner Violence” because it measured abusive experiences during the lifetime (both during childhood and adulthood). Therefore, we cannot determine when the abuse was experienced. In their cohort study of 200 women living with HIV, Knettel et al. (40) reported no association between experiences of abuse at any point in life and suicidal/self-harm ideation during pregnancy or at 6 months postpartum. However, it is difficult to evaluate the measure of abusive experiences because information about how this was assessed was omitted from the report.

#### Experiences of Abuse During Adulthood Only or Intimate Partner Violence

Similar to experiences of childhood abuse, recent experiences of IPV, particularly physical IPV, were shown to increase the likelihood of suicidal or self-harm ideation. Cross-sectional studies include Doi and Fujiwara (44) who found IPV during pregnancy increased odds of self-harm ideation over fourfold (verbal IPV) and almost fivefold (physical IPV) in a large sample of 5,960 postpartum women. The association was demonstrated in reverse by Iyengar et al. (47) who found those who reported suicidal ideation to be 10 times more likely to experience IPV in a smaller sample of 120 pregnant; however, details about the assessment of suicidal ideation are very limited in this study. In their “high” quality study of 426 women, Islam et al. (48) measured IPV during the first 6 months postpartum and found the odds of postpartum self-harm ideation were 2.65 times higher when a woman had experienced physical IPV following birth; however, neither sexual IPV nor psychological IPV experienced after birth affected the odds of self-harm ideation. Longitudinal findings have demonstrated a continuous effect of IPV during the perinatal period. According to Rodriguez et al. (49), having experienced physical IPV within the previous 4 weeks increased the odds of experiencing self-harm ideation during pregnancy and again at 12 months postpartum by 17% in a sample of 681 women. In their longitudinal study, Fisher et al. (50) used a modified version of the *EPDS* item 10 which asked “*I have had thoughts that I do not want to live any more*” to measure suicide ideation and noted that any form of lifetime IPV (emotional, sexual, or physical) significantly increased the odds of having thoughts of not wanting to live any more. Moreover, experiencing two or three forms of lifetime IPV was associated with almost eight times increased odds of suicide ideation. Aside from IPV, sexual trauma experienced during military service and self-harm ideation in women veterans in the United States were investigated by Gross et al. (51). In a sample of 620 women veterans, both military sexual harassment and trauma were significantly associated with self-harm ideation during pregnancy but not postpartum.

Previous studies also consistently found significant associations between experiences of abuse during adulthood and suicidal ideation and attempts, although the majority of studies was homogenous in design (i.e., cross-sectional) and conducted with pregnant women only. For example, in Asad et al.’s (52) sample of 1,369 pregnant women, 48% of participants had experienced verbal abuse and 20% had experienced physical or sexual abuse during the pregnancy or within the 6 months prior to the pregnancy. Asad et al. (52) conducted one of only three studies in this review (45, 52, 53) that provided separate data for suicide ideation and suicide attempts (rather than a combined measure of “suicidal behaviour”). Not only were there significant associations between experiences of any form of abuse and suicidal ideation and suicide attempts, but the frequency of the abuse was also associated with an increased likelihood of suicidal ideation and attempts. However, the authors did not find experiences of abuse to be more or less associated with suicidal ideation, nor more or less associated with suicide attempt. Current IPV was also associated with a sixfold increase in likelihood of suicidal ideation and attempts (a combined measure) in 214 pregnant women (54), and IPV experienced at any point during a woman’s lifetime was also found to be associated with increased odds of suicidal ideation, planning and attempts in a larger sample of 2062 pregnant women (45). According to Supraja et al.’s (55) bivariate analyses, any form of IPV (psychological, sexual, or physical) was strongly correlated with ideation during pregnancy, but IPV did not emerge as a significant predictor of ideation in their multivariate analysis. The authors asked participants who had attempted suicide during pregnancy to give a reason for their attempt and all eight of the women cited abuse and/or conflict with spouse as a reason for their attempt. Findings also demonstrated a link between history of rape and suicidal behaviour (56). In a sample of 988 women, Belete and Misgan (56) identified that an experience of rape increased the odds of suicidal behaviour by twofold at 6 weeks postpartum, although it is not clear whether “behaviour” refers to suicidal ideation, attempts, or both.

Two studies examined IPV and suicide deaths (57, 58). Both were case-control studies and used data extracted from the United States National Violent Death Reporting System whereby death by suicide was defined as a record of “death resulting from the intentional use of force against oneself.” Gold et al. (58) compared pregnant and postpartum women with women of reproductive age who were not pregnant or postpartum at the time of death. The authors identified a total of 2,083 female suicide victims of reproductive age from 17 US states between 2003 and 2007. Pregnant women who died by suicide were three times more likely to have experienced intimate partner conflict and postpartum women were over two times more likely to have experienced intimate partner conflict. Similarly, Adu et al. (57) used data gathered from 18 US states from 2003 to 2012 to compare suicides of pregnant and postpartum women with non-pregnant females (15–54 years) and then compared urban and rural differences. The authors identified a total of 4,306 female suicide victims and found recent intimate partner problems were associated with increased odds of the victim being pregnant or postpartum compared to non-perinatal. This observation supports Gold’s (58) findings and demonstrates that intimate partner problems as a key correlate for suicide during the perinatal period from 2007 to 2012. Two issues should be highlighted: a large proportion of Adu et al.’s (57) data were likely to have been the same data used in Gold et al.’s (58) study and neither study clarified what constituted an “intimate partner problem” or “conflict” and when and how frequently the “conflict” occurred.

To summarise, these studies consistently demonstrate that abuse experienced during adulthood increases the odds of suicide and self-harm ideation. There is also evidence that abuse experienced before and during the perinatal period was associated with ideation, and physical abuse appears to have the strongest association. IPV significantly increases odds of suicide planning, attempts and deaths, suggesting that receipt of abuse during adulthood cannot only trigger thoughts of suicide, but also enable women to act on those thoughts, and in some cases result in a fatal outcome.

#### Stress

Eleven studies measured risk factors that can be broadly grouped as “stress.” These risk factors include stressful life events experienced during pregnancy (59, 60) or experienced at any point in life (39, 53, 61), psychosocial stress during pregnancy (62), general perceived stress (63), pregnancy stress (61), parenting stress (64), stress about debt (65), post-traumatic stress symptoms (39, 66), and first-degree relative suicide (67). When grouped, these studies reported mixed findings with regards to stress and its association with ideation and behaviour. In Tavares et al.’s (60) unadjusted multivariate analysis, they found the prevalence of suicide ideation to be over four times greater in postpartum women who experienced two or more stressful life events during pregnancy, but this was no longer significant after adjustment for all significant correlates. The authors also failed to provide any information regarding which life events were considered stressful. It should also be noted that Tavares et al.’s (60) methodological quality was relatively poor (QATSDD rating of 45%). Gelabert et al. (59) used the *St Paul Ramsey Life Experience Scale* (68) to assess the impact of stressful life events, experienced during pregnancy, in six categories: primary support, social environment, housing, work, health, and economy. The authors treated stressful life events as a dichotomous variable whereby a score of at least two, in one or more of the categories, was classed as presence of stressful life events. Women who reported the presence of stressful life events were 88% more likely to experience self-harm ideation at some point during the first 32 weeks following childbirth (59). Gavin et al. (62) used the *Prenatal Psychosocial Profile* (69) which assesses the extent to which 11 events are causing stress, such as financial problems, feeling generally “overloaded” and current abuse. The authors found high levels of psychosocial stress experienced during pregnancy increased the odds of experiencing suicide ideation during pregnancy threefold. Focussing their investigations on parenting stress, Paris et al. (64) categorised a sample of 32 women with postpartum depression into “low” and “high” suicidality groups and noted that the high suicidality group perceived overall parenting as significantly more stressful.

With regards to suicidal ideation and attempts, Mezey et al. (53) reported data that show a significant association between lifetime exposure to traumatic events (e.g., life threatening illness, physical assault, imprisonment) and suicide ideation only and suicide attempts, in a sample of 200 pregnant and postpartum women. Palfreyman (65) assessed stress about debt specifically and found it was correlated with suicide ideation and behaviour in 1000 Sri Lankan pregnant women. With regards to posttraumatic stress symptoms, Maré et al. (39) reported those classed as “high risk” for suicidal ideation and behaviour were more likely to report post-traumatic stress than those who reported no suicidal ideation and behaviour in 748 pregnant and postpartum women.

#### Summary of Negative Life Events

Taken together, the studies (41–52, 54, 56–58) demonstrate that experiences of abuse, particularly physical abuse, are significantly associated with ideation and attempts when measured separately and collectively, and therefore may be involved in enabling a woman to transition from having suicidal thoughts alone to then act on those thoughts. Furthermore, the findings suggest a persistent psychological effect of abuse (e.g., lifetime IPV and childhood abuse) which can manifest during the perinatal period. There is some evidence to suggest experiencing stress during the perinatal period is associated with perinatal self-harm and suicide ideation and suicidal behaviour. However, the studies presented used different measures of perinatal stress and there was no consistency with regards to what constituted a stressful or traumatic life event, which limits the overall findings. Additionally, the life events measured may incorporate other psychosocial risk factors, such as IPV.

### Social Factors

Twenty-eight studies investigated social factors which were clustered into three groups of risk factors related to mother-infant bond/mother-infant interactions, social support, and loneliness. The methodological quality ratings of these 28 studies ranged from 36 to 86%, with 19 of the studies considered “good” quality, 7 “high,” and 2 “moderate.”

#### Mother-Infant Bond and Interactions

Takegata et al. (70) conducted a longitudinal study of three time points (during third trimester of pregnancy, 5 days postpartum, one month postpartum) with 243 women. They found those with self-harm ideation at any time point scored significantly higher on the “rejection and fear” and “anger and restrictedness” subscales of the *Japanese Postpartum Bonding Questionnaire* (*PBQ*) (71) at 5 days postpartum, and significantly higher on the “lack of affection,” “rejection and fear,” and “anger and restrictedness” *PBQ* subscales at one month postpartum. In another longitudinal study of 545 women, Gordon et al.’s (72) high quality study discovered that women who reported self-harm ideation at baseline (within 3 weeks of first antenatal midwifery appointment) had *PBQ* scores 6.28 points higher (i.e., poorer perceived bond) than those who did not report self-harm ideation. However, this difference was no longer significant after adjustment for depression at baseline. Furthermore, women who reported baseline self-harm ideation were significantly more controlling and infants significantly more compulsive in mother-infant interactions at 3 months postpartum. Two cross-sectional studies that assessed suicidal ideation report evidence of impaired mother-infant bonding and interactions. Faisal-Cury et al. (73) found bonding impairment was associated with almost five times increased odds of suicide ideation 6–9 months after birth, even when postpartum depressive symptoms were controlled for. Paris et al. (64) rated mother-infant interactions in 32 women with postpartum depression. Participants were categorised as “low suicidality” if they scored below 12 on the *Postpartum Depression Screening Scale* (*PDSS*) (74), or “high suicidality” if they scored 12 or higher on the *PDSS*. Compared to mothers identified as “low suicidality,” during unstructured interactions, the “high suicidality” mothers were significantly less aware of, and less able to consistently respond to, their infants’ social signals. Infants of the “high suicidality” mothers also exhibited less positive affect and more negative affect. However, the two groups behaved similarly in structured interactions (e.g., asking the parent to guide the infant to follow a rattle). In contrast, in a cohort of 430 women, Kubota et al. (75) did not observe any group difference in the mother-infant bond between those who did and did not report self-harm ideation. However, it should be noted that Kubota et al. (75) measured mother-infant bonding during early pregnancy (mean 5.3 months gestation) using the *Mother Infant Bonding Questionnaire* (76) which was designed to be used postnatally.

Martini et al. (46) investigated suicidal ideation and attempts and the mother-infant bond in a cohort of 306 mothers: mothers who reported perinatal suicidal behaviour indicated higher mean scores of bonding impairment with their infants compared to mothers with no perinatal suicidal behaviour, although these differences were not statistically significant.

#### Social Support

Twenty-four studies (28, 40, 43, 44, 46, 48, 54–56, 59–61, 65, 67, 75, 77–85) assessed social support, yet the types of social support (e.g., material, emotional, informational, affective, and social interaction) and sources of support (e.g., partner, family, friends, and neighbours) varied across these studies. With regards to the 16 studies that measured social support and suicide/self-harm ideation alone, seven studies found no association (48, 67, 80–82, 84, 85), six (40, 43, 44, 59–61) reported a significant association (i.e., poor social support increased the odds), and three (75, 78, 79) found a significant protective effect of social support (i.e., good-quality support reduced the odds). Both Doi and Fujiwara (44) and Tavares et al. (60) reported that a lack of social support was significantly associated with self-harm and suicide ideation, respectively, in their initial analyses but this association was no longer significant in their adjusted analyses. It should be noted that neither Doi and Fujiwara (44) nor Tavares et al. (60) provide information about the items used to assess support. In their longitudinal study, Gelabert et al. (59) measured functional social support (e.g., “*I have someone who goes to the doctor with me”*) and found that the women who reported self-harm ideation had significantly lower mean scores of functional social support at 2–3 days postpartum and at 8 weeks postpartum but no difference in scores at 32 weeks postpartum. Peltzer (79) used three items from the *Social Supportood Questionnaire* (86): “*If I were sick and needed someone to take me to a doctor, I would have trouble finding someone*,” “*I feel that there is no one I can share my most private concerns and fears*,” and “*I feel a strong emotional bond with at least one other person.*” In their sample of 580 postpartum women, the authors reported that the odds of self-harm ideation were reduced by 19% in women who perceived having social support, whilst being accompanied by the baby’s father to antenatal care was not found to affect the odds of self-harm ideation postpartum. In Giallo et al.’s (43) longitudinal study, a sample of 1,507 women at 12 months postpartum were asked if they would have liked more emotional support (e.g., someone to talk to about how they were feeling) over the past month. The sample were categorised into a “persistent self-harm ideation” class (38–43% probability of endorsing self-harm ideation across the study time point) and a “minimal self-harm ideation” class (1–3% probability of endorsing self-harm ideation across the study time points). Women who reported a need for emotional support at 12 months postpartum were almost twice as likely to be a member of the “persistent self-harm ideation” class.

Seven studies investigated social support and suicidal ideation and behaviour (46, 54–56, 65, 77, 83) with mixed findings, although it should be noted that most of these studies used a different self-report measure of social support. Only Belete and Misgan (56) and Belete et al. (77) used the same measure of social support, namely, the *Oslo-3 Social Support Scale* (87) which asks: “*how many people are you so close to that you can count on them if you have great personal problems?*,” “*how much interest and concern do people show in what you do?*,” and “*how easy is it to get practical help from neighbours if you should need it?.*” Belete and Misgan (56) assessed a sample of 988 postpartum women and did not find a significant association between social support and suicidal behaviour. However, in their sample of 738 pregnant women, Belete et al. (77) found that participants who reported poor social support were over three times more likely to indicate having seriously considered attempting suicide while pregnant. It is therefore unsurprising that Belete et al. (77) also reported that poor social support was correlated with suicide attempts during pregnancy. Onah et al. (83) used the *Multidimensional Scale of Perceived Social Support* (88), which consists of 12 items that measure closeness and emotional support from friends, family and a significant other (e.g., “*I can count on my friends when things go wrong*,” “*my family is willing to help me make decisions*,” “*there is a special person with whom I can share my joys and sorrows*”). In their sample of 376 pregnant women, the authors found that perceived support from a significant other did not have an effect on suicide ideation only, but perceived support was found to be associated with a reduced likelihood of suicide behaviour (defined by the authors as those who had suicidal thoughts and had proceeded to plan or prepare or attempt suicide). Martini et al.’s (46) longitudinal study used the *F-SozU* (89) which assesses general perceived or anticipated social support, excludes any support from healthcare professionals and includes items such as “*when I am sick, I can ask friends/relatives to handle important things for me without hesitation*” and “*there are people who accept me the way I am without reservations.*” In their cohort of 306 women, Martini et al. (46) reported that higher levels of perceived social support during pregnancy decreased the odds of perinatal suicidal behaviour, although ideation, planning and attempts were combined and therefore we cannot discern the specific effect of social support upon these unique outcomes.

As described previously, Gressier et al. (28) used the French Network of MBUs to retrospectively assess 1,439 women for suicide attempts and of these, 105 attempted suicide during the postpartum period and 49 had made attempts during pregnancy. Women who attempted suicide during pregnancy reported poor family/social support significantly more than those who attempted suicide when postpartum and those who did not attempt suicide during the perinatal period. However, no independent effect of family/social support on suicide attempt was observed in the logistic regression. The logistic regression did, however, reveal that tobacco and alcohol use significantly increased the odds of suicide attempts during pregnancy. The regression also identified history of miscarriage as having a significant independent association on suicide attempts during pregnancy. Gressier et al. (28) suggested that pregnancy ignited the previous trauma of losing a baby. Overall, Gressier et al.’s (28) results indicated that a mother’s distress had a greater effect on a woman’s inclination to act on suicidal thoughts than poor social support.

#### Loneliness

One study assessed loneliness: Kugbey et al. (54) failed to find a bivariate or multivariate association between loneliness and current suicidal behaviour (i.e., a composite score of suicidal ideation, suicide plans, and suicide attempts) in 214 pregnant women. However, they used just one item to measure loneliness without providing any more information on this item.

#### Summary of Social Factors

In the six studies that measured the mother-infant bond and/or interactions, the results start to advocate a link between the mother-infant relationship and ideation but not with suicidal behaviour. Several studies provided evidence that social support was important during the early postpartum period, when a woman appeared to be particularly vulnerable, and that emotional support demonstrated an association with self-harm ideation. Whilst the association between social support and ideation appears less strong, the studies that measured support and suicidal behaviour provided stronger evidence for an association. When viewed in combination, these 24 studies start to suggest that support may be more pertinent in the decision to act on thoughts rather than a trigger for the initial ideation.

### Cognitive Factors

A range of cognitive factors was investigated in seven studies (54, 63, 64, 82, 90–92). These studies’ methodological quality ratings ranged from 33 to 71%, with six of the seven studies considered “good” quality and one study was of “moderate” quality.

In a sample of 100 pregnant women, the association between decision-making function, assessed using the *Iowa Gambling Task* (93) and self-harm ideation was investigated (90). The participants were allocated to one of three groups: those who reported self-harm ideation, those who scored highly for depressive symptoms on the *EPDS* (33), but reported no self-harm ideation, and those who reported no self-harm ideation and did not score highly on the *EPDS*. Women in the self-harm ideation group demonstrated impaired decision-making compared to the other two groups in the fifth block of the task. Paris et al. (64) clustered a sample of 32 women with postpartum depression into “low” and “high” suicidality groups. When these two groups were compared, the mothers in the high suicidality group reported feeling significantly more emotionally labile, mentally confused and experienced a greater loss of self. Paris et al. (64) was the only study to investigate these cognitive factors and although the study employed a very small sample, it was rated high for methodological quality. Two studies explored rumination as a risk factor for perinatal self-harm ideation (63, 92) and both found negative perinatal-specific nocturnal rumination (i.e., having stressful thoughts about the pregnancy/infant while attempting to fall asleep) was associated with increased odds of self-harm ideation. In their more recent study, Kalmbach et al. (63) also found those who reported nocturnal cognitive hyperarousal (e.g., not being able to shut off thoughts when trying to fall asleep) at baseline were over 11 times more likely to report new onset self-harm ideation. However, this same study found perseverative thinking during the daytime was not associated with self-harm ideation, suggesting that cognitive-emotional dysregulation specifically at night plays a unique role in self-harm ideation.

Guilt, shame and worthlessness were assessed by three studies [guilt/shame (64); worthlessness/guilt (91); maltreatment-related shame (82)] and all three reported significant associations between their assessment of worthlessness/guilt/shame and suicidal ideation. Muzik et al. (82) identified maltreatment-related shame as the only risk factor in their logistic regression to be independently associated with the presence of suicidal ideation at 4 and 12 months postpartum; other factors included in the analysis, which did not demonstrate independent associations, were resilience, household income and marital status. As previously described, Paris et al. (64) categorised their sample as either “high suicidality” or “low suicidality.” On the guilt/shame subscale of the *PDSS* the “high suicidality” mothers scored significantly higher than the “low suicidality” mothers. Similarly, Benute et al. (91) divided their sample into those who reported suicidal ideation and those who did not and discovered that significantly more women in the suicidal ideation group endorsed feeling worthless/guilty; however, the authors only used one item to assess worthlessness/guilt.

### Personality and Individual Differences

Ten of the included studies assessed 16 different risk factors that could be categorised as personality and individual differences. Eight of the studies were rated as “good” quality and two were “high” quality with QATSDD scores ranging from 57 to 81%.

The association of self-esteem/self-efficacy was investigated with mixed results. Using the *Rosenberg Self-esteem Scale* (94), Islam et al. (48) reported high self-esteem to be protective against self-harm ideation postpartum, whereas Shi et al. (61) found those with self-harm ideation had relatively higher self-esteem, but self-esteem did not predict presence/absence of self-harm ideation before birth or postpartum. Paris et al. (64) used the *Maternal Self-Report Inventory-Short Form* (95) which assesses self-esteem and self-perceptions of parenting and motherhood over five domains: the mother’s perceived caretaking abilities, general ability and preparedness for motherhood, acceptance of her baby, expectations of a positive relationship with baby and feelings towards the birth. Mothers in the “high suicidality” group had lower total self-esteem scores, perceived they were less prepared for motherhood and expected a poorer relationship with their infants compared to those in the “low suicidality” group. Similarly, Bodnar-Deren et al. (80) investigated self-efficacy using five items that assessed perceived ability to care for the baby, other family members and the household. Self-efficacy showed a protective effect with almost 50% lower odds of suicide ideation in a sample of 1073 postpartum women.

Several personality traits were assessed by Takegata et al. (70) using the Japanese version of the *Temperament and Character Inventory* (96). In their cohort of 243 women, those who reported self-harm ideation at any time point (i.e., during the third trimester of pregnancy, 5 days postpartum or one month postpartum) had significantly lower mean scores for cooperativeness and self-directedness when compared to women who never reported self-harm ideation. Neither novelty-seeking, harm avoidance, persistence, reward-dependence nor self-transcendence demonstrated a significant association with self-harm ideation.

Neuroticism was assessed by three studies with mixed findings. Both Duan et al. (97) and Gelabert et al. (59) chose the *Eysenck Personality Questionnaire* (98) to measure neuroticism: Duan et al. (97) found neuroticism did not predict postpartum suicidal ideation, whereas Gelabert et al. (59) reported that neuroticism was associated with a marginal increase in the odds of self-harm ideation of 3% throughout the first 32 weeks following birth. Enătescu et al. (99) used the *NEO-Five Factor Inventory* (100) with a cohort of 202 women and reported higher levels of neuroticism in postnatal women with self-harm ideation than in those without, although neuroticism did not predict self-harm ideation.

Like neuroticism, there were mixed findings for an association between extraversion and self-harm ideation. Extraversion was negatively associated with self-harm ideation 2–3 days postpartum in a longitudinal study of 1795 women (59), whereas no differences in extraversion was found between those who reported self-harm ideation and those who did not, neither during pregnancy nor postpartum (99).

Only one study investigated the association between a personality/individual difference and suicide ideation and behaviour. In a sample of veterans, Szpunar et al. (66) did not find a significant correlation between hopelessness and suicidal behaviour. However, none of the participants reported a severe level of hopelessness and the lack of associations found may be due to the low variability of hopelessness reported and the very small sample of 28 women.

## Discussion

This review aimed to summarise the psychological and psychosocial risk factors associated with maternal suicide outcomes during the perinatal period and describe how these risk factors vary across self-harm and suicidal ideation, attempts and deaths. This was a comprehensive review of 59 studies making a novel contribution to the literature by synthesising findings of studies with a range of designs, conducted in low-, middle- and high-income countries with samples of both pregnant and postpartum women.

Most of the included studies used cross-sectional designs and therefore causal relationships could not be inferred. Regression models were commonly used to determine the shared relationships between the risk factors and suicide outcomes, and this paves the way for the use of alternative study designs and analyses in the future to investigate how apparently pertinent factors affect the trajectory of suicidal ideation and behaviour during the perinatal period. Only three studies measured suicide ideation and attempts separately and provided sufficient information to allow for a comparison of risk factors between these suicide outcomes (45, 52, 53), the other ten studies that investigated ideation and attempts either used a combined measure, arriving at one score for “suicidal behaviour” or did not provide sufficient data to make a comparison between the suicide outcomes. Comparing those women who experience suicidal ideation alone with those who have attempted suicide provides an opportunity to tease out which risk factors are unique and specific to suicide ideation or suicide attempts. It can be assumed that people who attempt suicide are also likely to experience suicidal ideation, and so only comparing those who experience suicidal ideation or attempt suicide with those who do not is no longer sufficient. Klonsky and May’s (101) ideation to action framework highlighted the need to better understand the distinct pathways and processes, i.e., what factors specifically contribute to the development of maternal suicide ideation, and then separately, what factors specifically promote the progression from ideation to maternal suicide attempts. That being said, all three studies that include specific measures of ideation and behaviour demonstrated the same risk factors for those with suicide ideation and those who had attempted suicide. Much of the identified research investigated psychosocial risk factors, such as negative life events and social factors, whereas there were far fewer studies that assessed cognitive factors and individual differences. This could be due to ease of measurement; for example, negative life events and social factors may be assessed with very few yes/no questions, whereas cognitive factors and personality traits require more complex validated measures. Furthermore, the psychological effects of negative life events and social factors have received greater research attention compared to those of the myriad cognitive factors and individual differences. It is important to bear in mind that many of the identified factors are not mutually exclusive. For example, IPV can cause long-term damage to the victim’s self-esteem (102), likewise feelings of guilt often persist into adulthood following abuse experienced during childhood (103). Therefore, determining pathways between psychological and psychosocial risk factors and suicidal behaviour in women is incredibly complex, but disentangling this web of risk factors is crucial for the development of assessments to identify and interventions to target maternal suicide ideation and behaviour.

To measure suicidal or self-harm ideation, 24 studies used the *EPDS* (33) item 10 which reads “*the thought of harming myself has occurred to me*” in the past 7 days, which may be interpreted in one of three ways: self-harm with suicide intent (i.e., suicide ideation), intentional self-harm with no suicide intent, or unintentional self-harm (e.g., accidentally falling down the stairs). Authors using this item often failed to provide a measure of severity of suicide/self-harm ideation. The widespread use is understandable because the *EPDS* is commonly used in primary care and maternity services to identify depressive disorders during pregnancy and the postpartum period (104). However, we cannot be sure of how respondents interpreted this item and whether self-harm with or without suicide intent was measured in the 24 studies. Current suicidal ideation screening instruments validated for use during the perinatal period, including the use of *EPDS* item 10, were primarily developed to assess maternal depression (25). It is therefore essential that future research efforts focus on designing a suicidal ideation measure for use during the perinatal period to assess suicidal thoughts, thoughts of self-harm with suicidal intentions and severity of suicidal thoughts.

### Negative Life Events

In multivariate models, experiences of IPV, particularly physical abuse, are associated with ideation, attempts and death by suicide, and the more frequently abuse is experienced, the greater the odds of ideation and attempt. This is interesting because the findings start to suggest that intimate partner abuse could trigger ideation and is then involved in a woman’s decision to act on those thoughts of self-harm and suicide. Similarly, childhood abuse was a strong predictor of both ideation and attempts. This dose-response relationship between adult and childhood trauma and suicide risk has been found by large-scale studies in the general population (e.g., 105, 106). There is also evidence to suggest that the relationship between depressive symptoms and IPV is bidirectional, with women who experience IPV at increased risk of depression, and women who report depressive symptoms being more likely to experience IPV, but curiously this finding has not been found in men (107). This might indicate the profundity of the effect of abuse on women. Experiencing violence as a perinatal woman may pose a particular risk because the stable and safe environment for her infant and herself, while she is very vulnerable, is threatened.

It is not necessarily surprising that abuse results in the victim feeling suicidal. Nevertheless, the findings included in this review solidify that abuse experienced in childhood and adulthood is prevalent around the world and that the effects can persist and manifest during this important period of a woman and infant’s life. It is essential for suicide prevention during the perinatal period to identify and help women who have experienced or are currently experiencing abuse. The volume of studies and the strength of the findings also reinforce the need to identify which psychological factors are most affected when abuse is experienced, which could then help with the development of an intervention to target these mediating psychological mechanisms in order to reduce suicidal thoughts and behaviour.

### Social Factors

The review findings suggest that support may play a lesser part in the development of suicidal ideation but provides some evidence that lack of support is associated with suicidal behaviour. Lack of support appeared to be particularly risky during pregnancy, which could demonstrate that women are especially aware of the support around them during pregnancy, fearful if there is no support network in place for the baby’s arrival, and of being unsupported when coping with the major changes a baby will bring. Previous research has demonstrated that lack of social support is significantly associated with postnatal depression and health-related quality of life (108). Reid and Taylor (109) found that support from an intimate partner and from friends and family was protective against postpartum depression but was insufficient to reduce the effect of exposure to stress (e.g., sexual assault, IPV, parenting-related stress), found to be significantly associated with postpartum depression. Qualitative research conducted with women living with HIV and perinatal depression reported that women described social support as being composed of interaction, encouragement, and “offloading”/sharing worries (110). Interaction served as a distraction from their worries, encouragement was helpful to alleviate depressive feelings because it gave women strength, and “offloading” was viewed as an opportunity for women to alert others of their suicidal thoughts (110). Not only do these findings highlight the importance of social support for perinatal women because it alleviated perinatal depression using different functions, but it also identified offloading as particularly critical for preventing maternal suicide.

The review identified some evidence to suggest an association between self-harm and suicidal ideation and the mother-infant bond and interactions. An association between poor mother-infant bonding and postpartum depressive symptoms has been well-documented (e.g., 111, 112). However, how bonding contributes to maternal mood and how depressive symptoms contribute to poor bonding is not well understood. Understandably, the mother-infant bond does not feature in models of suicidal behaviour based on research conducted with the general population, further highlighting the need to develop a model of suicidal behaviour specific to the perinatal period.

### Cognitive Factors

A variety of cognitive factors were investigated. Decision-making was found to be impaired in those who reported self-harm ideation after five blocks of the decision-making task, suggesting an impairment only occurs over time and/or after making many decisions. Interestingly, one study suggests impaired decision-making may influence the occurrence of interpersonal problems and increase the risk of problematic affective relationships. Worthlessness, guilt, emotional lability, mental confusion, loss of self, emotional lability, and shame were found to be associated with suicide and self-harm ideation. Feeling worthless is a common symptom of depression (113, 114) and has been shown to predict lifetime suicide attempts in those who have experienced serious trauma (e.g., a physical or sexual assault) but not in those who have not experienced trauma (115). This finding suggests that worthlessness can mediate the relationship between negative life events (e.g., IPV and childhood abuse) and suicidal behaviour. With regards to loss of self, there is very limited empirical research that investigates this factor and suicide. However, qualitative research has linked “loss of former identity” to postnatal depression (116).

### Personality and Individual Differences

The one study that investigated hopelessness, failed to find an association with suicidal ideation and behaviour, although the sample reported low variability of hopelessness (66). Interestingly, findings in the general population suggest that hopelessness is involved in the development of suicidal ideation but it is not useful for predicting the transition from ideation to suicide attempts and death (32).

Self-esteem, defined as one’s judgement of self-worth and others’ judgement of oneself (117), and self-efficacy, defined as one’s perceived ability and motivation (117), were both investigated as risk factors for suicide. Findings start to suggest that high levels of self-esteem and self-efficacy could be protective and low levels could be a risk for self-harm and suicide ideation. In psychiatric outpatients, other-based self-esteem (i.e., beliefs about others’ judgement of oneself) was found to be the strongest predictor of suicidal ideation after controlling for depression and hopelessness (118). The postpartum period is a time of learning new skills while developing the new identity of “mother” and it is therefore unsurprising that a woman’s perceived ability to be a mother and care for her infant, or self-efficacy, could impact on a mother’s mental wellbeing. Previous research has identified that support from family and friends (119) and marital support (120) are positively associated with maternal self-efficacy postpartum and negatively associated with maternal depression. Although risk factors were treated as separate entities for the purposes of this review, these associations between support and postpartum self-efficacy highlight the interconnectedness of the risk factors for maternal suicide, and in turn, the complex nature of teasing out the causal and mediational relationships.

### Strengths and Limitations of the Review

This novel review is the first to focus on psychological and psychosocial factors rather than include any or all correlates of maternal suicide, many of which cannot be targeted by psychological interventions. Strengths of this review include the use of a prospective protocol and adherence to the PRISMA statement to ensure the review was conducted and the findings reported in a transparent manner, as well as no limitations on language and date of publication, which resulted in a comprehensive and thorough review of studies conducted around the world. Furthermore, an independent reviewer also conducted reliability checks to ensure rigour of study screening, selection, data extraction and quality appraisal of the identified studies.

The review is somewhat limited by the narrative, rather than statistical, synthesis of results. Most of the risk factors were investigated by few studies that employed heterogenous designs and therefore a meta-analysis would not have been suitable for the majority of risk factors. The intentionally inclusive sampling strategy resulted in the inclusion of studies with wide ranging samples, such as veterans (51, 66) and HIV-positive women (40, 49, 79, 121, 122). Although it is important that suicidal ideation and behaviours are investigated in all groups of women, this may have resulted in the inclusion of some risk factors that will not apply to all women, such as military sexual trauma. Finally, only two papers investigated suicide deaths (57, 58) and these two studies only investigated IPV. It is therefore difficult to draw conclusion about the impact of different risk factors on this most severe suicide outcome.

### Implications and Future Research

Four implications for identification of women at increased risk of suicide and potential methods for intervening to reduce suicidal ideation and behaviour in clinical practice are evident. The review findings suggest it is very important that perinatal women who have experienced domestic violence whether it be from an intimate partner or another member of the household, or childhood abuse, are identified. Therefore, ensuring midwives routinely ask women about childhood abuse and domestic violence during the booking appointment is the first clinical implication of this research. Secondly, those who indicate they have had these negative experiences should be helped to leave the abuse if ongoing and offered psychological support. The occurrence of negative life events cannot be modified, but the psychological sequelae could be, therefore future research should aim to determine which psychological factors are affected by abuse and how they trigger or sustain suicidal ideation and behaviour. The review also suggests ensuring women have emotional and practical support, whether it be from an intimate partner or friends and family, especially during pregnancy, may help to reduce the likelihood of suicidal behaviour. Therefore, ensuring midwives help women identify their supportive network during pregnancy offers a third clinical implication. Fourthly, midwives should also facilitate a mother to strengthen her access to support if required.

The theoretical basis for which psychological and psychosocial factors are involved in the development of suicidal ideation and behaviour is derived from research with the general population. We know that the perinatal period is unique in that women are facing many new changes and challenges and that perinatal suicides differ to non-perinatal suicides [e.g., perinatal suicides often occur through more violent means (13–15)]. Thus, to establish psychological and psychosocial factors involved in perinatal suicide, research needs to focus specifically on perinatal samples.

Many of these correlational findings are mixed and do not consider that women’s situations vary dramatically, therefore missing the more important nuances that could impact on a woman’s risk of suicide. Qualitative approaches offer more flexibility and scope to investigate what could lead a woman to feeling suicidal during the perinatal period and help untangle the web of interconnected risk factors. There are many other psychological factors known to be associated with suicide risk in other populations, such as impulsivity and optimism (32), which have not been assessed by any of the included studies. Future qualitative research could also investigate potential risk factors which have not been investigated previously in the perinatal population.

## Conclusion

This novel review was the first to focus on psychological and psychosocial factors rather than include any or all correlates of maternal suicide, many of which cannot be targeted by psychological interventions. There was strong evidence to indicate that abuse, either experienced recently or during childhood, is associated with suicide ideation, attempted suicide and death. There was also convincing evidence that a lack of social support is particularly important during the perinatal period and was significantly associated with suicidal behaviour. Clinically, investigating the role of psychological and psychosocial factors in the development of suicidal ideation and behaviour is essential to the generation of assessments and interventions to identify and reduce maternal suicidal ideation and behaviour.

## Data Availability Statement

The original contributions presented in the study are included in the article/Supplementary Material, further inquiries can be directed to the corresponding author.

## Author Contributions

HR, AW, and DP developed the initial idea for this review. HR undertook the systematic review, under the supervision of AW, DP, and DE. HR wrote the first draft of the manuscript. All authors contributed to the article and have approved the final manuscript.

## Conflict of Interest

The authors declare that the research was conducted in the absence of any commercial or financial relationships that could be construed as a potential conflict of interest.

## Publisher’s Note

All claims expressed in this article are solely those of the authors and do not necessarily represent those of their affiliated organizations, or those of the publisher, the editors and the reviewers. Any product that may be evaluated in this article, or claim that may be made by its manufacturer, is not guaranteed or endorsed by the publisher.

## Appendix A. Medline Search

**TABLE 2:** The search strategy for MEDLINE.

1	exp SUICIDE
2	exp SUICIDAL IDEATION
3	exp SUICIDE, ATTEMPTED
4	exp SELF-INJURIOUS BEHAVIOR
5	(suicid* OR parasuicid* OR self*harm* OR self-injur* OR self-inflict* OR poisoning OR drug overdose OR self-poison* OR overdose OR self mutilat*).ti,ab.
6	1 OR 2 OR 3 OR 4 OR 5
7	exp MOTHERS
8	exp WOMEN
9	exp PREGNANCY
10	exp POSTPARTUM PERIOD
11	exp PERIPARTUM PERIOD
12	exp PERINATAL DEATH
13	(mother* OR pregnan* OR antenatal OR postpartum OR peripartum OR perinatal OR postnatal OR puerper*).ti,ab.
14	7 OR 8 OR 9 OR 10 OR 11 OR 12 OR 13
15	6 AND 1
