# Supplementary material for: Maternal Suicide Ideation and Behaviour During Pregnancy and the First Postpartum Year: A Systematic Review of Psychological and Psychosocial Risk Factors
Source: Front Psychiatry. 2022 Mar 24;13:765118. doi: 10.3389/fpsyt.2022.765118 (PMC8987004; doi:10.3389/fpsyt.2022.765118)
Supplement: Supplementary file 1 [file Table_1.docx]

| **Supplementary Table 1. Study characteristics**  *Note*. Papers were ordered first by suicide variable (suicidal/self-harm ideation only 🡪 suicidal/self-harm ideation and suicide attempt/self-harm 🡪 suicide attempt only 🡪 suicide death), then by study design (case-control 🡪 cohort 🡪 cross-sectional), then by year of publication and then in alphabetical order. | | | | | | | | | |
| --- | --- | --- | --- | --- | --- | --- | --- | --- | --- |
| **No.** | **Authors, date and location** | **Research aims** | **Design** | **Sample**  **(n;**  **perinatal status;**  **age;**  **ethnicity;**  **parity;**  **relationship status;**  **education;**  **diagnosis)** | **Suicide variable (measure)** | **Psychological/ psychosocial variable (measure)** | **Analysis** | **Main findings** | **Limitations** |
| **Suicidal/self-harm ideation only** | | | | | | | | | |
| 1 | Bao et al. (90)  2021  China | - determine if sleep quality and decision-making ability are associated with suicidal ideation in pregnant women with depression compared to pregnant women without depression | Case-control | - n=100;  - all pregnant;  - mean 28.9 years;  - all Han Chinese;  - 54% nulliparous;  - all married;  - mean 13 years of education;  - excluded women with mental illness and/or chronic disease | SHI  (EPDS item 10) | Decision-making (IGT) | Bivariate analysis | As the IGT progressed, the decision-making ability of those with SHI was significantly worse than those with no SHI but high EPDS score and worse than those with no SHI and a low EPDS score. SH  I negatively correlated with decision-making ability. | Use of single item to measure suicide variable. |
| 2 | Leeners, Rath, Block, Görres & Tschudin (42)  2014  Germany | - investigate obstetrical risk factors for unfavourable pregnancy outcome in women exposed to adverse and abusive childhood experiences;  - compare perinatal outcomes in women exposed and not exposed to abusive experiences;  - analyse the association between childhood abuse and obstetrical risk factors | Case-control | - n=255;  - all pregnant;  - mean 27.1 years;  - 99% Caucasian;  - limited parity info;  - 84% married/stable relationship;  - no education info;  - no diagnosis info | SI  (yes/no) | Childhood sexual abuse (regular unstructured interviews, items adapted from WSHQ); Childhood physical abuse (regular unstructured interviews); Adverse childhood experiences (regular unstructured interviews); Abuse during pregnancy (yes/no) | Bivariate analysis & logistic regression | Physical childhood abuse was associated with increased odds of SI during pregnancy. | Use of single item to measure SI and limited information about the item. |
| 3 | Kalmbach, Ahmedani, Gelaye, Cheng & Drake (63)  2021  USA | - prospectively investigate clinical insomnia, nocturnal cognitive hyperarousal, and nocturnal perinatal-focused rumination as predictors of SI in perinatal depression | Cohort | - n = 39;  - T1: 30 weeks gestation  - T2: 31 weeks gestation  - T3: 32 weeks gestation  - T4: 33 weeks gestation…  - T17: 6-8 weeks postpartum;  - mean 28.2 years;  - 41% White;  - 56% multiparous;  - no relationship info;  - no education info;  - no diagnosis info | SHI (EPDS item 10) | Nocturnal cognitive hyperarousal (PSAS-C); Nocturnal perinatal-specific rumination (1 item developed by authors); Perseverative thinking (PTQ); Perceived stress (PSS) | Logistic regression | The odds of SHI increased by almost 8-fold when endorsing nocturnal cognitive hyperarousal and almost 6-fold when reporting perinatal-focused rumination. The odds of women reporting new onset SHI increased over 11-fold when they reported baseline nocturnal cognitive hyperarousal. Perseverative thinking and perceived stress were not independently associated with SHI. | Use of single item to measure suicide variable. |
| 4 | Enătescu et al. (99)  2020  Romania | - identify the presence of SI among pregnant women and postpartum women;  - explore the personality traits correlated with an increased likelihood of developing SI in perinatal women | Cohort | - n=202;  - T1: third trimester of pregnancy  - T2: 6-8 weeks postpartum;  - mean 28.5 years  - no ethnicity info;  - no parity info;  - 98% married/stable relationship;  - 85% Lyceum/ University;  - no diagnosis info | SHI  (EPDS item 10) | Neuroticism, extraversion, openness to experience, agreeableness, conscientiousness (NEO-FFI) | Bivariate analysis & logistic regression | Agreeableness was associated with increased odds of SHI during pregnancy and conscientiousness was associated with reduced odds of SHI during pregnancy. | Use of single item to measure suicide variable. |
| 5 | Gelabert et al. (59)  2020  Spain | - examine the prevalence of postpartum SI;  - study the role of personality dimensions as risk factors of postpartum SI, considering depression, psychiatry history, social support, and stressful life events | Cohort | - n=1795;  - T1: 2-3 days postpartum  - T2: 8 weeks postpartum  - T3: 32 weeks postpartum;  - mean 31.8 years;  - all Caucasian;  - 47% primiparous;  - 97% married/steady partner;  - 28% college degree;  - 17% personal psychiatric history | SHI  (EPDS item 10) | Neuroticism, extraversion, psychoticism (EPQ-RS); Social support (Duke-UNC FSSQ); Stressful life events during pregnancy (St. Paul Ramsey LES) | Bivariate analysis & logistic regression | Neuroticism, psychoticism and stressful life events during pregnancy predicted postpartum SHI. | Use of single item to measure suicide variable. |
| 6 | Gross, Kroll-Desrosiers & Mattocks (51)  2020  USA | - inform perinatal mental health after military sexual trauma | Cohort | - n=620  - T1: 20-32 weeks gestation  - T2: 8-24 weeks postpartum  - mean 33.2 years  - 60% White;  - no parity info;  - 65% married;  - no education info;  - 57% depression, 42% post-traumatic stress disorder, 47% anxiety disorder, 12% mood disorder, 7% attention deficit hyperactivity disorder, 7% bipolar disorder, 4% eating disorder | SHI  (EPDS item 10) | Military sexual trauma (VHA MST) | Bivariate analysis & mediation analysis | Military sexual trauma (both harassment and assault) was significantly associated with SHI during pregnancy. | Use of single item to measure suicide variable. |
| 7 | Knettel et al. (40)  2020  Tanzania | - examine the course and correlates of SI among women living with HIV during pregnancy and at 6 months postpartum | Cohort | - n=200;  - T1: pregnancy  - T2: 6 months postpartum;  - mean 30 years;  - no ethnicity info;  - no parity info;  - 85% married/in a relationship;  - 59% ≤ primary school;  - all HIV positive | SHI & SI (EPDS item 10 and PHQ item 9) | General social support (PAS); Social support from infant’s father (NSSQ); Interpersonal violence (childhood abuse and/or IPV; no measure info) | Logistic regression | Low social support and low support from the infant’s father were significantly associated with ideation. | Measures were administered in-person and therefore responses may have been subject to social desirability bias. |
| 8 | Kubota et al. (75)  2020  Japan | - elucidate the risk factors for SI among perinatal women | Cohort | - n=430;  - T1: early pregnancy  - T2: late pregnancy  - T3: 5 days postpartum  - T4: 1 month postpartum;  - mean 33 years;  - no ethnicity info;  - 82% nulliparous;  - no relationship info;  - mean 15 years of education;  - 17% presence of mental disorder | SHI  (EPDS item 10) | Mother-infant bonding (MIBQ); Social support (SSQ-12) | Bivariate analysis & logistic regression | Good quality social support in early pregnancy was significantly associated with reduced odds of SHI. Mother-infant bonding showed no association with SHI. | Mother-infant bonding was assessed during early pregnancy. |
| 9 | Gordon et al. (72)  2019  UK | - investigate the association between maternal self-harm (lifetime history of self-harm and self-harm ideation during pregnancy) and mother-infant interactions | Cohort | - n=545;  - T1: within 3 weeks of first antenatal visit  - T2: 28 weeks gestation  - T3: 3 months postpartum;  - 52% White;  - no parity info;  - 87% married/ partnered;  - 60% University/ professional qualification;  - no diagnosis info | SHI  (EPDS item 10) | Mother-infant bonding (PBQ); Mother-infant interactions (CARE-Index) | Linear regression | Women wo reported SHI during pregnancy had a lower perceived mother-infant bond. In mother-infant interactions, SHI was associated with maternal controlling behaviours and infant compulsive behaviours. | Use of single item to measure suicide variable. |
| 10 | Jones, Rodriguez, Alcaide, Weiss & Peltzer (121)  2019  South Africa | - compare rates of SI at 12 months postpartum in HIVV-infected women with detectable versus undetectable efavirenz during pregnancy | Cohort | - n=599;  - T1: prior to 24 weeks gestation  - T2: 32 weeks gestation  - T3: 12 months postpartum;  - mean 28.7 years;  - no ethnicity info;  - no parity info;  - 54% unmarried and living separately;  - 32% ≥ Grade 12 achieved;  - all HIV positive | SHI  (EPDS item 10) | IPV during past 4 weeks (CTS-18) | Bivariate analysis & logistic regression | The odds of postpartum SHI were significantly higher among women who reported antenatal psychological IPV. | Use of single item to measure suicide variable. |
| 11 | Takegata, Takeda, Sakanashi, Tanaka & Kitamura (70)  2019  Japan | - identify group(s) of women during the perinatal period with thoughts of self-harm;  - clarify the psychological and clinical correlates including personality traits of self-harm ideation | Cohort | - n=243;  - T1: third trimester of pregnancy  - T2: 5 days postpartum  - T3: 1 month postpartum;  - mean 30.1 years;  - no ethnicity info;  - limited parity info;  - 98% married;  - no education info;  - no diagnosis info | SHI  (EPDS item 10) | Novelty seeking, harm avoidance, reward dependence, persistence, self-directedness, cooperativeness, self-transcendence (TCI);  Mother-infant bonding (PBQ) | Cluster analysis | Low self-directedness, low cooperativeness and lack of affection and anger and rejection towards the baby were associated with women who reported SHI. | Use of single item to measure suicide variable. |
| 12 | Giallo et al. (43)  2018  Australia | - investigate the extent to which women report self-harm ideation from pregnancy to 4-years postpartum;  - identify groups of women defined by their trajectories of self-harm ideation from early pregnancy to 4-years postpartum;  - identify preconception and early postnatal factors associated with women's trajectories of self-harm ideation | Cohort | - n=1507;  - T1: 10-24 weeks gestation  - T2: 3 months postpartum  - T3: 6 months postpartum  - T4: 12 months postpartum  - T5: 18 months postpartum  - T6: 4 years postpartum;  - 18-46 years;  - no ethnicity info;  - all nulliparous;  - 58% married;  - 69% ≤12 years education;  - no diagnosis info | SHI  (EPDS item 10) | Childhood abuse (CMHSR);  Fear of partner (yes/no); Perceived need for emotional support (yes/no);  IPV during first postpartum year (CAS) | Latent class analysis & logistic regression | Childhood physical abuse and perceived lack of emotional support were associated with endorsing persistent SHI. | Use of single item to measure suicide variable. |
| 13 | Rodriguez et al. (49)  2018  South Africa | - identify risk factors for SI in women living with HIV during pregnancy;  - investigate the trajectory of SI into the postnatal period and the associated factors | Cohort | - n=681;  - T1: 8-24 weeks gestation  - T2: 32 weeks gestation  - T3: 6 months postpartum  - T4: 12 months postpartum;  - mean 28.5 years;  - no ethnicity info;  - 20% nulliparous;  - 59% single;  - 72% <12years education;  - all HIV positive | SHI  (EPDS item 10) | IPV during past 4 weeks (CTS-18) | Bivariate analysis & logistic regression | Physical IPV increased the odds of experiencing SHI during pregnancy and at 12 months postpartum by 17%. | Use of single item to measure suicide variable. |
| 14 | Shi, Ren, Li & Dai (61)  2018  China | - observe depression and SI at immediate prenatal (1 week before childbirth) and postpartum stages (3-7 days postpartum) and the causal relationship between them;  - explore the psychosocial risk or protective factors of depression and SI | Cohort | - n=213;  - T1: late pregnancy, during hospital admission for childbirth  - T2: 3-7 days postpartum;  - 20-42 years;  - all Chinese;  - 83% nulliparous;  - all married;  - 60% ≥University;  - no diagnosis info | SHI  (EPDS item 10) | Self-esteem (RSET); Life events (LESPW); Pregnancy stress (PPS); Social support (SSS) | Bivariate analysis & logistic regression | Women with prenatal SHI had higher self-esteem and lower social support. Women with postpartum SHI perceived more stress and experienced more life events during pregnancy. | Use of single item to measure suicide variable. |
| 15 | Bodnar-Deren, Klipstein, Fersh, Shemesh & Howell (80)  2016  USA | - investigate the prevalence of SI in the first 6 months postpartum and identify baseline sociodemographic, clinical and psychosocial characteristics associated with later postpartum SI;  - examine whether self-report symptoms of depression and/or anxiety assessed 24-48 hours after delivery were associated with later postpartum SI | Cohort | - n=1073;  - T1: 24-48 hours postpartum  - T2: 3 weeks postpartum  - T3: 3 months postpartum  - T4: 6 months postpartum;  - mean 30.2 years;  - 44% White;  - 44% primiparous;  - 80% married;  - 70% >high school;  - 19% past history of depression | SHI & SI (EPDS item 10 and PHQ item 9) | Social and partner support (7 items developed by authors); Self-efficacy (5 items developed by authors) | Bivariate analysis & logistic regression | Increased self-efficacy was associated with lower odds of ideation. | Baseline data were collected at 1-2 days postpartum so may be confounded by postpartum blues. |
| 16 | Muzik, Brier, Menke, Davis & Sexton (82)  2016  USA | - describe the point and period prevalence and severity of SI from 4 to 18 months postpartum;  - examine risk/ protective factors for SI among postpartum women with child maltreatment histories;  - identify whether these risk/protective factors had differential relationships to SI at specific time points postpartum | Cohort | - n=116;  - T1: 4 months postpartum  - T2: 6 months postpartum  - T3: 12 months postpartum  - T4: 15 months postpartum  - T5: 18 months postpartum;  - mean 29.5 years;  - 65% Caucasian;  - no parity info;  - 66% married;  - 75% >high school;  - no diagnosis info | SI  (PDSS) | Childhood abuse severity (CTQ); Maltreatment-related shame (SAQ); Perceived support (FAPGAR); Resilience (CD-RISC) | Bivariate analysis, linear regression & logistic regression | Shame was associated with SI presence at 4 and 12 months postpartum. Resilience was associated with SI severity at 4 months postpartum and social support was associated with SI severity at 6 months postpartum. | Cross-sectional regression analysis does not allow for identification of factors associated with trajectories of SI risk. |
| 17 | Fisher et al. (50)  2013  Vietnam | - establish the prevalence of different forms of IPV experienced by women during their lifetimes and in the perinatal period;  - describe the socio-demographic characteristics of women who experienced IPV;  - examine the associations between different forms of IPV and women’s mental health during pregnancy and after childbirth in rural Vietnam | Cohort | - n=495;  - T1: 12-20 weeks gestation  - T2: at least 28 weeks gestation  - T3: 8 weeks postpartum  - T4: 6 months postpartum;  - mean 26.1 years;  - no ethnicity info;  - 41% nulliparous;  - all married;  - 81% ≥grade 6;  - no diagnosis info | SI  (Modified EPDS item 10) | Lifetime IPV (WHO VAW) | Logistic regression | Any form of lifetime IPV was associated with increased odds of SI. Experiencing two or three forms of lifetime IPV was associated with almost eight times increased odds of SI. | Duration of relationship was not assessed and therefore cannot establish whether IPV occurred in current relationship. |
| 18 | Crandall, Sridharan & Schermer (123)  2010  USA | - determine whether modifiable societal risk factors would contribute to thoughts of death among depressed, disadvantaged mothers;  – determine whether depression and thoughts of death would affect maternal and infant health outcomes | Cohort | - n=704;  - T1: childbirth-related hospital stay  - T2: 1 year postpartum  - T3: 3 years postpartum;  - mean 26.3 years;  - 40% African American;  - no parity info;  - no marital status info;  - 58% graduated high school;  - all met criteria for depression | SI  (WHO CIDI short form) | IPV (no measure info) | Bivariate analysis & logistic regression | Physical IPV was associated with 60% increased odds of SI. | Two-item screen used for identification of SI may underestimate the incidence of SI. |
| 19 | Abdelghani et al. (124)  2021  Egypt | - evaluate the demographic, obstetric and psychosocial correlates of current suicide risk among pregnant women in Egypt | Cross-sectional | - n=835;  - all pregnant;  - mean 28.7 years;  - no ethnicity info;  - 38% nulliparous;  - all in a relationship;  - 88% ‘educated’;  - no diagnosis info | SI (BSS) | Lifetime IPV (yes/no) | Bivariate analysis & logistic regression | Lifetime IPV was associated with almost 9 times increased odds of SI. | Cross-sectional and therefore causal explanations of IPV and SI cannot be made. |
| 20 | Faisal-Cury, Levy & Matijasevich (73)  2021  Brazil | - estimate the prevalence of SI in postpartum mothers who had antenatal depression  - evaluate the association between bonding impairment and SI | Cross-sectional | - n=358;  - all postpartum;  - mean 26.5 years;  - 30% White;  - 35% primiparous;  - 83% married/cohabiting;  - 19% <9 years education;  - no diagnosis info | SI  (PHQ item 9) | Mother-infant bonding (PBQ) | Bivariate analysis & logistic regression | Bonding impairment was associated with over four times increased odds of SI. | Use of single item to measure SI. |
| 21 | Zewdu, Reta, Yigzaw & Tamirat (85)  2021  Ethiopia | - determine the magnitude of SI in Gondar town among HIV-positive perinatal women | Cross-sectional | - n=414;  - all pregnant;  - 60% 26-33 years;  - no ethnicity info;  - 37% multiparous;  - 73% married;  - 24% ≥ diploma  - all HIV positive | SI (no measure info) | Social support (Oslo-3 SSS) | Bivariate analysis & logistic regression | No association between ‘moderate and above’ social support and SI was found. | No information as to how SI was measured. |
| 22 | Akram, Ahmed, Maqsood & Bibi (78)  2020  Pakistan | - investigate the relationship between postpartum depression, perceived social support and SI | Cross-sectional | - n=547;  - all postpartum;  - all 22-33 years;  - no ethnicity info;  - all primiparous;  - no relationship info;  - no education info;  - 50% diagnosed with hearing loss | SI  (SIDAS) | Perceived support (MSPSS) | Logistic regression & mediation analysis | Perceived support was a significant negative predictor of SI among new mothers with hearing loss. | Relatives of mothers with hearing loss helped to communicate scale items to the mothers and this may have influenced the mothers responses to items, especially those regarding social support. |
| 23 | Iyengar, Bondade & Raj (47)  2020  India | - find out the prevalence of IPV and associated psychiatric comorbidities in pregnant women | Cross-sectional | - n=120;  - all pregnant;  - mean 25.2 years;  - no ethnicity info;  - no parity info;  - all married;  - 82.5% ≥ high school;  - 18% anaemia, 15% hypothyroidism, 7% hypertension, 3% other physical illness | SI  (no measure info) | IPV (WHO VAW) | Bivariate analysis & logistic regression | In women who had SI, the odds of experiencing IPV were increased 10-fold. | No information as to how SI was measured. |
| 24 | Kalmbach et al. (92)  2020  USA | - explore the associations among sleep symptoms, nocturnal rumination (general and perinatal-specific), depressive symptoms, and SI | Cross-sectional | - n=267;  - all pregnant;  - mean 29.8 years;  - 56% White;  - 36% nulliparous;  - 90% married/in a relationship;  - no education info;  - 5% gestational diabetes, 2% pre-eclampsia, 3% hypertension | SHI  (EPDS item 10) | Nocturnal general rumination (PSAS-C); Nocturnal perinatal-specific rumination (2 items developed by authors) | Bivariate analysis & logistic regression | High negative perinatal-specific rumination was associated with over 3 times increased odds of SHI. | Use of single item to measure suicide variable. |
| 25 | Zhang et al. (125)  2020  China | - examine the association between childhood abuse, including emotional abuse, physical abuse, and sexual abuse, and SI among pregnant women in the general population in China | Cross-sectional | - n=1825;  - all pregnant;  - 77% < 35 years;  - 99% Han Chinese;  - 46% nulliparous;  - 99.6% married;  - 81% ≥ junior college;  - 1% history of mental health disorders | SI  (PHQ item 9) | Childhood abuse (CTQ) | Logistic regression | Women who experienced any form of childhood abuse (physical, sexual, emotional) were almost two and half times more likely to have SI than those who experienced no childhood abuse. | Use of single item to measure SI. |
| 26 | Doi & Fujiwara (44)  2019  Japan | - examine the combined effect of maternal adverse childhood experiences and maternal age on self-harm ideation among postpartum women | Cross-sectional | - n=5960;  - all postpartum;  - 37% 30-34 years;  - no ethnicity info;  - 49% primiparous;  - 98% married;  - 34% completed college;  - no diagnosis info | SHI  (EPDS item 10) | Adverse childhood experiences (items developed by authors);  IPV during pregnancy (items developed by authors);  Social support (items developed by authors) | Logistic regression | Postpartum women with 3 or more adverse childhood experiences and younger age (<25 years) were 10 times more likely than those with no ACEs and older age (35 years+) to have SHI. | Use of single item to measure suicide variable. |
| 27 | Duan et al. (97)  2019  China | - investigate the association between trait neuroticism and SI in postpartum women;  - explore whether anxiety and depression mediate the association | Cross-sectional | - n=1027;  - all postpartum;  - mean 29.9 years;  - 99% Han Chinese;  - 48% primiparous;  - 97% married;  - 76% ≥ Bachelor degree;  - no diagnosis info | SI  (PHQ item 9) | Neuroticism (Neuroticism subscale of EPQ) | Logistic regression & mediation analysis | Trait neuroticism did not predict SI directly in the postpartum period. Women with high neuroticism were prone to increased anxiety and depression which in turn, indirectly increased the risk of SI. | Use of single item to measure SI. |
| 28 | Islam et al. (48)  2019  Bangladesh | - to examine the association of experiencing IPV after childbirth on postpartum SI;  - examine whether postpartum depression and self-esteem act to mediate the association | Cross-sectional | - n=426;  - all postpartum;  - 44% 19-24 years;  - no ethnicity info;  - 41% primiparous;  - all married;  - 67% ≥ secondary and higher education;  - no diagnosis info | SHI  (EPDS item 10) | IPV since childbirth (WHO VAW);  Self-esteem (RSET);  Childhood sexual abuse (yes/no);  Social support (FNS-SSS) | Bivariate analysis & logistic regression | The odds of postpartum SHI were significantly higher among women who reported physical IPV following childbirth and high self-esteem significantly reduced reports of SHI. | Use of single item to measure suicide variable. |
| 29 | Rurangirwa, Mogren, Ntaganira, Govender & Krantz (126)  2018  Rwanda | - assess the prevalence of non-psychotic mental health disorders in postpartum women;  - study the association between different forms of IPV exposure during pregnancy and non-psychotic mental health disorders | Cross-sectional | - n=921;  - all postpartum;  - 15-47 years;  - no ethnicity info;  - no parity info;  - 84% married/ cohabiting;  - 50% not completed primary school;  - 21% major depressive episode, 20% generalised anxiety disorder, 8% post-traumatic stress disorder | SI  (MINI) | IPV during pregnancy (20 items from WHO WHLEQ) | Logistic regression | Physical IPV, sexual IPV, psychological IPV and controlling behaviour were strongly associated with SI. | Measured IPV during pregnancy but measured SI 1-14 months postpartum. |
| 30 | Tabb et al. (84)  2018  Brazil | - identify whether exposure to IPV was associated with increased risk for SI among low-income postpartum women from São Paulo, Brazil | Cross-sectional | - n=701;  - all postpartum;  - 16-44 years;  - 47% White;  - no parity info;  - 73% married/ cohabiting;  - 54% ≥9 years education;  - 28% postpartum depression, 14% past psychiatric treatment | SI  (1 item from CIS-R) | IPV during postpartum (adapted WHO VAW); Social support (MOS) | Poisson regression | Postpartum IPV was associated with three times increased risk of SI. | Use of single item to measure SI. |
| 31 | Rodriguez, Cook, Peltzer & Jones (122)  2017  South Africa | - estimate the prevalence of and identify risk factors for SI among pregnant women living with HIV in rural South Africa | Cross-sectional | - n=673;  - all pregnant;  - mean 28.4 years;  - no ethnicity info;  - 21% nulliparous; 41% married/ cohabiting;  - 71% <12years education;  - all HIV positive | SHI  (EPDS item 10) | IPV during past week (CTS) | Bivariate analysis & logistic regression | Physical IPV during pregnancy was associated with increased odds of SHI. | Use of single item to measure suicide variable. |
| 32 | Castro e Couto et al. (81)  2016  Brazil | - evaluate the prevalence of suicidality during the second trimester of pregnancy;  - ascertain whether certain sociodemographic, obstetric, psychosocial and clinical risk factors are associated with suicidality | Cross-sectional | - n=255;  - all pregnant;  - mean 28.0 years;  - 73% non-Caucasian;  - 48% nulliparous;  - 71% married;  - 56% >10 years education;  - 7% lifetime bipolar disorder, 38% anxiety disorder, 1% psychosis, 17% antenatal depression | SHI & SI (MINI Plus, EPDS item 10 and BDI item 9) | Prenatal support (items developed by authors); IPV (items developed by authors) | Univariate analysis & logistic regression | Psychosocial risk factors investigated did not result in any significant differences. | Sample contained a proportion of adolescents that might have overestimated suicidality risk. |
| 33 | Shamu, Zarowsky, Roelens, Temmerman & Abrahams (127)  2016  Zimbabwe | - investigate the association of postnatal depression and SI with emotional, physical and sexual IPV experience by women during pregnancy | Cross-sectional | - n=842;  - all postpartum;  - 44% 15-25 years;  - no ethnicity info;  - no parity info;  - 88% married;  - 93% >primary education;  - 21% postpartum depression | Lifetime SI (yes/no), SI during past 4 weeks (yes/no) | IPV during pregnancy (WHO VAW); Childhood abuse (items developed by authors) | Univariate analysis & logistic regression | Three or more events of emotional IPV during pregnancy was associated with almost two and a half times increased odds of SI. | Cross-sectional and therefore causal explanations of IPV and SI cannot be made. |
| 34 | Zhong et al. (128)  2016  Peru | - examine the association between exposure to childhood abuse and SI among pregnant women | Cross-sectional | - n=2964;  - all pregnant;  - mean 28.1 years;  - 75% Mestizo;  - 49% nulliparous;  - 81% married/ cohabiting;  - 96% ≥7 years education;  - no diagnosis info | SI  (PHQ item 9) | Childhood abuse (CPSAQ); Lifetime IPV (items adapted from WHO VAW and items adapted from DVM) | Bivariate analysis & logistic regression | Any childhood abuse was associated with almost 3 times increased odds of SI, even with adjustment for lifetime IPV and depressive symptoms. | Use of single item to measure SI. |
| 35 | Alhusen, Frohman & Purcell (129)  2015  USA | - examine the prevalence of SI and comorbid depressive symptomology during pregnancy;  - identify the risk factors for SI in a low-income sample of pregnant women. | Cross-sectional | - n=166;  - all pregnant;  - mean 23.3 years;  - 93% Black;  - no parity info;  - 54% single;  - 66% <12years education;  - no diagnosis info | SHI  (EPDS item 10) | IPV during pregnancy  (AAS) | Bivariate analysis & logistic regression | IPV during pregnancy was associated with over nine times increased odds of SHI. | Use of single item to measure suicide variable. |
| 36 | Fonseca-Machado, Alves, Haas, Monteiro & Gomes-Sponholz (130)  2015  Brazil | - investigate the association between IPV and indicators of SI during pregnancy | Cross-sectional | - n=358;  - all pregnant;  - mean 25.0 years;  - 62% non-White;  - 60% multiparous;  - 80% cohabiting;  - 11% ≥12 years education;  - no diagnosis info | SI  (BSS) | Lifetime IPV & IPV during pregnancy (WHO VAW) | Logistic regression | IPV during pregnancy was associated with over six times increased odds of SI. | Cross-sectional and therefore causal explanations of IPV and SI cannot be made. |
| 37 | Peltzer (79)  2015  South Africa | - investigate the prevalence of SI and its associated factors among postpartum women living with HIV in South Africa | Cross-sectional | - n=580;  - all postpartum;  - mean 28.6 years;  - no ethnicity info;  - no parity info;  - 29% married/ cohabiting;  - 36% ≥grade 12;  - all HIV positive | SHI  (EPDS item 10) | IPV during past 12 months (no measure info); Social support (3 items from SSQ) | Bivariate analysis & logistic regression | Higher perceived social support decreased the odds of SHI. | Use of single item to measure suicide variable. |
| 38 | Sit et al. (41)  2015  USA | - examine associations between SI and plausible risk factors (trauma history, sleep disturbance, and anxiety symptoms) in depressed postpartum women | Cross-sectional | - n=628;  - all postpartum;  - mean 28.7 years;  - 73% White;  - 63% multiparous;  - 56% married/ cohabiting;  - 73% >high school;  - 90.4% depressive disorders | SHI  (EPDS item 10) | Childhood physical & sexual abuse (DDIS); Adult physical & sexual abuse (DDIS) | Bivariate analysis & logistic regression | Childhood physical abuse was associated with increased odds of SHI. Childhood sexual abuse was not significantly associated with SHI. | Use of single item to measure suicide variable. |
| 39 | Farias et al. (67)  2013  Brazil | - describe the prevalence of psychiatric disorders and to identify the factors associated with current suicide risk in the first trimester of pregnancy | Cross-sectional | - n=239;  - all pregnant;  - mean 26.7 years;  - 75% non-White;  - 22% ≥2 children;  - 79% married/stable relationship;  - 58% ≥9 years education;  - 15% major depression disorder, 17% agoraphobia, 11% generalised anxiety disorder | SI  (MINI) | IPV (CTS-1); Social support (MOS); First-degree relative suicide (yes/no) | Poisson regression | Prevalence of SI is 2-fold greater when women suffered physical IPV. | Cross-sectional and therefore causal explanations of IPV, social support and SI cannot be made. |
| 40 | Tavares et al. (60)  2012  Brazil | - evaluate the prevalence of suicide risk and comorbidities in postpartum women | Cross-sectional | - n=919;  - all postpartum;  - 70% 20-34 years;  - no ethnicity info;  - no parity info;  - 74% cohabiting;  - 32% completed high school;  - 21% mood disorders, 3% panic disorder, 5% social phobia, 4% generalised anxiety disorder, 5% obsessive-compulsive disorder, 4% post-traumatic stress disorder | SI  (MINI) | Social support (no measure info); Stressful life events during pregnancy (no measure info) | Poisson regression | Crude analysis showed poor social support and experiencing stressful events during pregnancy were associated with increased SI. Adjusted analysis did not show that these factors were associated with SI. | Women who were diagnosed with depression during pregnancy were referred to psychiatric services. Data relating to any treatment women received were not collected. |
| 41 | Benute et al. (91)  2011  Brazil | - identify the risk of suicidal behaviour in high-risk pregnant women in Brazil | Cross-sectional | - n=268;  - all pregnant;  - 46% 19-30 years;  - no ethnicity info;  - no parity info;  - 73% with partner;  - 7% >secondary school;  - 10% depression | SI  (PHQ item 9) | Worthlessness or guilt feelings (PHQ-9) | Bivariate analysis | Worthlessness/ feelings of guilt were significantly positively correlated with SI. | Use of single item to measure SI. |
| 42 | Gavin, Tabb, Melville, Guo & Katon (62)  2011  USA | - examine the prevalence of SI and comorbid psychiatric disorders during pregnancy;  - identify the risk factors for SI during pregnancy | Cross-sectional | - n=2159;  - all pregnant;  - mean 30.6 years;  - 66% White;  - 40% nulliparous;  - 87% cohabiting;  - 80% >12 years education;  - 5% probable antenatal depression, 3% probable panic disorder | SI  (PHQ item 9) | Psychosocial stress during pregnancy (PPP); IPV during past 12 months (no measure info) | Bivariate analysis & logistic regression | High levels of psychosocial stress was associated with over three times increased odds of SI. | Use of single item to measure SI. |
| 43 | Paris, Bolton & Weinberg (64)  2009  USA | - determine the prevalence of SI in mothers with postpartum depression;  - determine how mothers who score lower or higher on SI appear on measures assessing emotional and cognitive functioning, maternal self-esteem and parenting stress;  - determine whether mothers who are more suicidal appear different to observers on ratings of mother-infant interactions | Cross-sectional | - n=32;  - all postpartum;  - mean 32.5 years;  - 78% Caucasian;  - 72% primiparous;  - 91% married/ partnered;  - 60% graduate degree;  - 53% recently started taking medication for depression | SI  (PDSS) | Emotional lability (PDSS); Loss of self (PDSS); Guilt/shame (PDSS); Mental confusion (PDSS); Self-esteem (MSI-SF); Parenting stress (PSI-SF); Mother-infant interactions (CIB) | Bivariate analysis | The high suicidality group (when compared to the low suicidality group) perceived parenting as significantly more stressful, had lower self-esteem, felt more emotionally labile, guilty/shameful and mentally confused, and experienced a greater loss of self. | Very small sample size, limited power to go beyond bivariate analysis. |
| **Suicidal/self-harm ideation and suicide attempt** | | | | | | | | | |
| 44 | Maré et al. (39)  2021  South Africa | - assess the prenatal and postnatal prevalence and correlates of SI and behaviour via stratified analyses of SI and behaviour presence and severity | Cohort/  Cross-sectional (n=121 seen at both time points) | - n=748;  - T1: 28-32 weeks gestation  - T2: 6 months postpartum;  - median 25.5 years;  - no ethnicity info;  - 64% multiparous;  - 59% single;  - 65% completed primary and some secondary school;  - 24% HIV positive | SI & SA (MINI) | Childhood abuse (CTQ); Stressful life events (TLEQ);  IPV (measure developed by authors); Posttraumatic stress symptoms (MPSS) | Bivariate analysis & logistic regression | Those who experienced recent IPV were over twice as likely to report postpartum SI and SA. Those with ‘low-risk’ SI and SA were more likely to experience recent IPV and childhood abuse compared to those with no SI and SA. Those with ‘high risk’ SI and SA were more likely to have posttraumatic stress symptoms compared to those with no SI and SA. | Longitudinal analysis not possible due to the small number of participants seen at both time points. |
| 45 | Szpunar, Crawford, Baca & Lang (66)  2020  USA | - examine the feasibility of recruiting pregnant veterans into a longitudinal study;  - characterize hopelessness, depression, posttraumatic stress symptoms and suicidality in this population | Cohort | - n=28;  - T1: 3rd trimester of pregnancy  - T2: 6 weeks postpartum;  - mean 31.6 years;  - 46% Caucasian;  - 39% primiparous;  - 68% married/  cohabiting;  - 54% ≥ Bachelor degree;  - no diagnosis info | Recent SI & SA (C-SSRS);  Lifetime SI & SA (C-SSRS) | Hopelessness (BHS); Posttraumatic stress symptoms (PCL-5) | Bivariate analysis | Posttraumatic stress symptoms were not significantly correlated with SI during pregnancy or postpartum. | Very small sample size, limited power to go beyond bivariate analysis. |
| 46 | Martini et al. (46)  2019  Germany | - investigate predictors of perinatal SI and behaviour;  - examine features of the mother-child relationship in mother-infant dyads with vs. without perinatal SI and behaviour | Cohort | - n=306;  - T1: 10-12 weeks gestation  - T2: 22-24 weeks gestation  - T3: 35-37 weeks gestation  - T4: 10 days postpartum  - T5: 2 months postpartum  - T6: 4 months postpartum  - T7: 16 months postpartum;  - mean 28.2 years;  - no ethnicity info;  - 59% primiparous;  - 94% cohabiting;  - 69% >10 years education;  - 8% panic disorder, 7% agoraphobia, 3% social phobia, 32% any phobia, 12% generalised anxiety disorder, 3% obsessive-compulsive disorder, 7% posttraumatic stress disorder, 14% perinatal depression, 40% perinatal anxiety | SHI (EPDS item 10);  SI (WHO CIDI-V items E36, E37, E37a and BSI item 9);  SA (WHO CIDI-V item E38) | Childhood abuse/rape (WHO CIDI-V);  Mother-infant bonding (PBQ); Social support (F-SozU) | Logistic regression | Social support significantly reduced the odds of SI and behaviour. Although not statistically significant, women who were suicidal indicated higher mean scores of impaired bonding. | The small number of women who indicated SI and behaviour limits the statistical power. |
| 47 | Belete, Kassew, Demilew & Zeleke (77)  2021  Ethiopia | - assess the prevalence and factors associated with SI and SA among pregnant women | Cross-sectional | - n=738;  - all pregnant;  - mean 25.5 years;  - no ethnicity info;  - 19% nulliparous;  - 89% married;  - 14% no formal education;  - 1% history of diagnosed mental disorder, 7% chronic medical illness | SI (WHO CIDI);  SA (WHO CIDI) | Social support (Oslo-3 SSS);  Lifetime and current IPV (WHO VAW) | Bivariate analysis & logistic regression | Poor social support was associated with over three times increased odds of SI. Social support was also significantly correlated with SA. | Conducted during the Covid-19 pandemic and only included women attending hospital for antenatal care – limited generalizability because many women will have been unable to attend due to Covid-19-related restrictions. |
| 48 | Kugbey et al. (54)  2021  Ghana | - examine the prevalence and correlates of prenatal depression, anxiety, and current suicidal behaviours among pregnant women in the Volta Region of Ghana | Cross-sectional | - n=214;  - all pregnant;  - 55% 26-35 years;  - no ethnicity info;  - no parity info;  - 67% married;  - no education info;  - no diagnosis info | SI & SA (items developed by authors) | Loneliness (item developed by authors); Body image satisfaction (item developed by authors); Childhood abuse (item developed by authors); Lifetime IPV (item developed by authors); Current IPV (item developed by authors); Partner support (item developed by authors); Family support (item developed by authors); Friend support (item developed by authors) | Bivariate analysis & logistic regression | Women who reported current IPV were over 6 times more likely to report SI and SA. | Data do not exclusively represent SI/SA during the perinatal period. |
| 49 | Palfreyman (65)  2021  Sri Lanka | - investigate the prevalence of lifetime- and current-pregnancy SI and/or behaviour  - investigate the relationship between depression and SI and behaviour in pregnant women  - identify correlates of depression and SI and behaviour | Cross-sectional | - n=1000;  - all pregnant;  - mean 28 years;  - 60% Sinhalese;  - 46% primiparous;  - 97% married;  - 78% completed primary and some secondary school;  - no diagnosis info | SI & SA (C-SSRS) | Social support (measure developed by authors); Stress about debt (measure developed by authors); Physical IPV during pregnancy (measure developed by authors); IPV in current relationship (measure developed by authors); Justification for IPV experienced (measure developed by authors) | Bivariate analysis & logistic regression | SI and SA during pregnancy was associated with stress from debt, perceived lack of social support, justifying IPV and all forms of IPV. Women who experienced at least one form of IPV in their current relationship were over four times more likely to report SI and SA during pregnancy. | Cross-sectional and therefore causal explanations of IPV and SI and behaviour cannot be made. |
| 50 | Belete & Misgan (56)  2019  Ethiopia | - determine the prevalence and the associated factors of suicidal behaviour among postnatal mothers | Cross-sectional | - n=988;  - all postpartum;  - 62% 25-34 years;  - no ethnicity info;  - 46% primiparous;  - 82% married;  - 84% literate;  - 8% history of depression | SI (MINI);  SA (MINI); | Social support (Oslo-3 SSS); Lifetime and current abuse (AAS); History of rape (items developed by authors) | Bivariate analysis & logistic regression | Mother with a history of rape were at significantly higher risk for suicidal behaviour (SI, suicide planning and SA). | Cross-sectional and therefore causal explanations of abuse, rape, social support and suicidal behaviour cannot be made. |
| 51 | Levey et al. (45)  2019  Peru | - characterise suicidal behaviour among a cohort of pregnant women;  - identify risk factors for transitions between suicidal behaviours | Cross-sectional | - n=2062;  - all pregnant;  - mean 27.9 years;  - 80% Mestizo;  - 46% nulliparous;  - 82% married/ cohabiting;  - 55% >12 years education;  - no diagnosis info | SI (Suicide Questionnaire from WHO CIDI);  SA ( Suicide Questionnaire from WHO CIDI) | Childhood abuse (yes/no);  IPV during lifetime (yes/no) | Logistic regression | Childhood abuse and lifetime IPV were associated with increased odds of SI, suicide planning and SA. | Data do not exclusively represent SI/SA during the perinatal period. |
| 52 | Onah, Field, Bantjes & Honikman (83)  2017  South Africa | - investigate the prevalence, associated risk and comorbidities of SI and behaviour among pregnant women in low-income women in urban South Africa | Cross-sectional | - n=376;  - all pregnant;  - 69% 18-29 years;  - no ethnicity info;  - limited parity info;  - 39% married;  - 60% >grade 10;  - limited diagnoses info | SI during pregnancy (MINI Plus); Lifetime SA (MINI Plus) | Perceived support (MSPSS); IPV during past 12 months (CTS-2) | Bivariate analysis & logistic regression | Increased perception of social support reduced odds of SI and suicidal behaviour. IPV was associated with two times increased odds of SI and suicidal behaviour. | Data collected from women attending an obstetric unit, may miss the most vulnerable women. |
| 53 | Supraja et al. (55)  2016  India | - determine the prevalence of SI, planning and SAs during early pregnancy;  - identify associated and predictive factors of SI, planning and SAs among women attending a public health antenatal clinic | Cross-sectional | - n=462;  - all pregnant;  - mean 23.0 years;  - no ethnicity info;  - 51% multiparous;  - all married;  - 72% ≤8 years education;  - no diagnosis info | SHI (EPDS item 10);  SI and SA during pregnancy (modified SBQ-R);  Lifetime SI and SA (modified SBQ-R) | IPV (ICMR); Social support (MSSS) | Bivariate analysis & logistic regression | Any form of IPV was strongly correlated with SI. Higher perceived social support decreased the odds of SI. Reasons for SA during pregnancy included conflict with spouse and in-laws. | Results may be specific to low-income urban women. |
| 54 | Asad et al. (52)  2010  Pakistan | - determine the prevalence of suicidal thoughts and SAs;  - identify demographic variables and mental health correlates of suicidal thoughts and SA among pregnant women in urban Pakistan | Cross-sectional | - n=1369;  - all pregnant;  - 65% ≥25 years;  - no ethnicity info;  - no parity info;  - no marital status info;  - 84% some formal/informal education;  - 18% depression and/or anxiety | SI (1 item from AKUADS-SF and items developed by authors);  SA (items developed by authors) | Abuse during pregnancy (items developed by authors) | Logistic regression | Verbal abuse was associated with over four times increased odds of SI and almost four times increased odds of lifetime SA. Physical/sexual abuse was associated with over two times increased odds of SI and almost three times increased odds of lifetime SA. | Cross-sectional and therefore causal explanations of abuse, SI and SA cannot be made. |
| 55 | Mezey, Bacchus, Bewley & White (53)  2005  UK | - examine the prevalence and types of traumatic events, reported by women and the impact of the traumatic events on women's mental health, particularly with respect to posttraumatic stress disorder and depression | Cross-sectional | - n=200;  - 47% antepartum, 53% postpartum;  - mean 30.6 years;  - 56% White;  - no parity info;  - 80% married/ cohabiting;  - no education info;  - 18% depression | SI (yes/no);  SA (yes/no) | Lifetime IPV (AAS); Lifetime exposure to traumatic events (PTDS) | Logistic regression | A relationship between exposure to traumatic events and SI was found and a relationship between exposure to traumatic events and SA was found. | Very little info about SI and SA measures. |
| 56 | Farber, Herbert & Reviere (38)  1996  Location not specified | - examine whether a history of childhood physical or sexual abuse is related to increased risk for suicidality during pregnancy | Cross-sectional | - n=309;  - all pregnant;  - mean 27.3 years;  - 93% African American;  - no parity info;  - 15% married;  - no education info;  - 25% depressive disorder/adjustment disorder with depressed mood, 6% anxiety disorder, 3% psychotic disorder | SI during pregnancy (no measure info);  Lifetime SA (no measure info) | Childhood abuse (no measure info) | Bivariate analysis | A significantly greater percentage of women who experienced childhood physical abuse reported SI. A significantly greater percentage of women who experienced childhood sexual abuse reported SI and lifetime SA. | Data do not directly address history of abuse as a factor in SAs during pregnancy, rather lifetime SA was investigated. |
| **Suicide attempt** | | | | | | | | | |
| 57 | Gressier et al. (28)  2017  France & Belgium | - identify risk factors for SAs in pregnancy and/or in the postpartum period among women with a psychiatric disorder hospitalized in the year following childbirth in a mother and baby unit | Cross-sectional | - n=1439;  - all postpartum;  - mean 31.3 years;  - no ethnicity info;  - no parity info;  - 76% married;  - 55% >12 years education;  - 5% anxiety disorder, 29% psychosis, 14% major depressive episode, 17% recurrent depression, 21% bipolar disorder, 11% personality disorder, 4% addictive disorder | SA  (reported by participant or recorded in medical files) | Childhood sexual abuse (MCC); Childhood maltreatment (MCC); Foster care in childhood (MCC); Child’s father support (MCC); Family/ social support (MCC) | Bivariate tanalysis & logistic regression | Poor family/social support was significantly more frequent when SA occurred in pregnancy but was found not to be significantly associated with SA. | Data collected from women admitted to mother and baby units, may miss the most vulnerable women. |
| **Suicide death** | | | | | | | | | |
| 58 | Adu, Brown, Asaolu & Sanderson (57)  2019  USA | - examine whether rural-urban county status modifies the association between suicide risk factors and pregnancy status among pregnant, postpartum and non-pregnant female suicide for the years 2003 to 2012 | Case-control | - n=4306;  - reproductive-age females (3% pregnant, 3% postpartum);  - limited age info;  - limited ethnicity info;  - no parity info;  - limited relationship status info;  - limited education info;  - limited diagnoses info | SD  (recorded as ‘death resulting from the intentional use of force against oneself’ in the US NVDRS) | Intimate partner problem (US NVDRS record) | Bivariate analysis & logistic regression | Intimate partner problems were associated with increased odds that the suicide victim was pregnant or postpartum compared to non-pregnant. | Info regarding intimate partner problems are obtained from the reports of families/friends of the deceased without independent verification and may be subject to lack of relevant knowledge, response bias, and recall bias. |
| 59 | Gold, Singh, Marcus & Palladino (58)  2012  USA | - examine victims of suicides during pregnancy, suicides up to one year postpartum and non-pregnancy-associated suicides  - compare psychiatric history, substance use, methods of suicide, intimate partner problems, and precipitating circumstances among these groups | Case-control | - n=2083;  - reproductive-age females (2% pregnant, 3% postpartum);  - pregnant mean 31.0 years, postpartum mean 32.0 years, non-pregnant/postpartum mean 40.0 years;  - limited ethnicity info;  - no parity info;  - >33% married;  - limited education info;  - 95% mood disorders, 9% anxiety disorder, 5% schizophrenia | SD  (recorded as ‘death resulting from the intentional use of force against oneself’ in the US NVDRS) | Conflict with intimate partner (US NVDRS record) | Bivariate analysis & logistic regression | Pregnant women who died by suicide had three times increased odds of intimate partner conflict. Postpartum women who died by suicide had over two times increased odds of intimate partner conflict. | Women who are pregnant/ postpartum are more likely to have had interactions with an intimate partner, so findings may be related to greater prevalence of relationship in these women as opposed to non-pregnant/postpartum women. |
| IPV = intimate partner violence; SA= suicide attempts; SD = suicide deaths; SHI = self-harm ideation; SI = suicidal ideation; US NVDRS = United States National Violent Death Reporting System  Measures abbreviations, AAS = Abuse Assessment Screen; AKUADS-SF = Aga Khan University Anxiety and Depression Scale – Short Form; BDI = Beck Depression Inventory; BHS = Beck Hopelessness Scale; BSI = Brief Symptom Inventory; BSS = Beck Scale for Suicide Ideation; CARE-Index = Child-Adult Relationship Experiment Index; CAS = Composite Abuse Scale; CD-RISC = Connor-Davidson Resiliency Scale; CIB = Coding Interactive Behaviour manual; CIS-R = Clinical Interview Schedule – Revised; CMHSR = Child Maltreatment History Self Report; CPSAQ = Childhood Physical and Sexual Abuse Questionnaire; C-SSRS = Columbia-Suicide Severity Rating Scale; CTQ = Childhood Trauma Questionnaire; CTS = Conflict Tactics Scale; DDIS = Dissociative Disorders Interview Schedule; Duke-UNC FSSQ = Duke-UNC Functional Social Support Questionnaire; DVM = Demographic Health Survey Questionnaires and Modules: Domestic Violence Module; EPDS = Edinburgh Postnatal Depression Scale; EPQ = Eysenck Personality Questionnaire; EPQ-RS = Eysenck Personality Questionnaire – Revised Short Scale; FAPGAR = Family Adaptation, Partnership, Growth, Affection and Resolve Scale; FNS-SSS = Family Needs Screener Social Support Scale; F-SozU = Fragebogen zur sozialen Unterstützung Social Support Questionnaire; ICMR = Indian Council of Medical Research Task Force Study on Domestic Partner Violence Questionnaire; IGT = Iowa Gambling Task; LESPW = Life Events Scale for Pregnant Women; MCC = Marcé Clinical Checklist; MINI = Mini International Neuropsychiatric Interview; MOS = Medical Outcomes Study Social Support Survey; MPSS = Modified Posttraumatic Stress Disorder Symptom Scale; MSI-SF = Maternal Self-Report Inventory – Short Form; MSPSS = Multidimensional Scale of Perceived Social Support; MSSS = Maternity Social Support Scale; NEO-FFI = NEO Five-Factor Inventory; NSSQ = Norbeck Social Support Questionnaire; PAS = Perceived Availability of Support Scale; PBQ = Postpartum Bonding Questionnaire; PCL-5 = Posttraumatic Stress Disorder Checklist for DSM-5; PDSS = Postpartum Depression Screening Scale; PHQ = Patient Health Questionnaire; PPP = Prenatal Psychosocial Profile; PPS = Pregnancy Pressure Scale; PSAS-C = Presleep Arousal Scale – Cognitive Factor; PSI-SF = Parenting Stress Index – Short Form; PSS = Perceived Stress Scale; PTDS = Posttraumatic Diagnostic Scale; PTQ = Perseverative Thinking Questionnaire; RSET = Rosenberg Self-esteem Test; SAQ = Shame Attributions Questionnaire; SBQ-R = Suicide Behaviours Questionnaire – Revised; SSQ = Social Support Questionnaire; SSS = Social Support Scale; St. Paul Ramsey LES = St. Paul Ramsey Life Experience Scale; TCI = Temperament and Character Inventory; TLEQ = World Mental Health Traumatic Life Events Questionnaire; VHA MST = Veterans Health Administration Military Sexual Trauma Screening Instrument; WHO CIDI = World Health Organization Composite International Diagnostic Interview; WHO VAW = World Health Organization Violence Against Women Instrument; WHO WHLEQ = World Health Organisation Women’s Health and Life Experiences Questionnaire; WSHQ = Wyatt Sexual History Questionnaire | | | | | | | | | |
