# Supplementary material for: Maternal Suicide Ideation and Behaviour During Pregnancy and the First Postpartum Year: A Systematic Review of Psychological and Psychosocial Risk Factors
Source: Front Psychiatry. 2022 Mar 24;13:765118. doi: 10.3389/fpsyt.2022.765118 (PMC8987004; doi:10.3389/fpsyt.2022.765118)
Supplement: Supplementary file 2 [file Table_2.docx]

| **Supplementary Table 2. Study quality ratings (QATSDD scores)** | | | | | | | | | | | | | | | | | | | | | | | | | | | | | | | | | | | |
| --- | --- | --- | --- | --- | --- | --- | --- | --- | --- | --- | --- | --- | --- | --- | --- | --- | --- | --- | --- | --- | --- | --- | --- | --- | --- | --- | --- | --- | --- | --- | --- | --- | --- | --- | --- |
| **QATSDD criteria** | | | **1) Explicit theoretical framework (/3)** | **2) Statement of aims/ objectives in main body of report (/3)** | **3) Clear description of research setting (/3)** | | **4) Evidence of sample size considered in terms of analysis (/3)** | **5) Representative sample of target group of a reasonable size (/3)** | | **6) Description of procedure for data collection (/3)** | | **7) Rationale for choice of data collection tool(s) (/3)** | | | **8) Detailed recruitment data (/3)** | | **9) Statistical assessment of reliability and validity of measurement tool(s) (Quantitative only) (/3)** | | **10) Fit between stated research question and method of data collection (Quantitative only) (/3)** | | | **11) Fit between stated research question and format and content of data collection tool e.g. interview schedule (Qualitative only)** | | | **12) Fit between research question and method of analysis (/3)** | **13) Good justification for analytical method selected (/3)** | | **14) Assessment of reliability of analytical process (Qualitative only)** | | **15) Evidence of user involvement in design (/3)** | | **16) Strengths and limitations critically discussed (/3)** | | **Total (/42)** | **%** |
| **Suicide and self-harm ideation** | | | | | | | | | | | | | | | | | | | | | | | | | | | | | | | | | | | |
| 1 | Bao et al. (90) | | 3 | 3 | 3 | | 0 | 2 | | 1 | | 0 | | | 0 | | 0 | | 2 | | |  | | | 3 | 3 | |  | | 0 | | 2 | | 22 | 52 |
| 2 | Leeners, Rath, Block, Görres & Tschudin (42) | | 3 | 3 | 3 | | 3 | 3 | | 2 | | 0 | | | 3 | | 0 | | 3 | | |  | | | 3 | 2 | |  | | 2 | | 3 | | 33 | 79 |
| 3 | Kalmbach, Ahmedani, Gelaye, Cheng & Drake (63) | | 2 | 3 | 3 | | 0 | 2 | | 2 | | 0 | | | 1 | | 0 | | 2 | | |  | | | 3 | 2 | |  | | 0 | | 1 | | 21 | 50 |
| 4 | Enătescu et al. (99) | | 2 | 3 | 3 | | 0 | 2 | | 2 | | 1 | | | 2 | | 1 | | 2 | | |  | | | 3 | 3 | |  | | 0 | | 2 | | 26 | 62 |
| 5 | Gelabert et al. (59) | | 3 | 3 | 3 | | 0 | 3 | | 2 | | 0 | | | 3 | | 0 | | 2 | | |  | | | 3 | 3 | |  | | 0 | | 3 | | 28 | 67 |
| 6 | Gross, Kroll-Desrosiers & Mattocks (51) | | 2 | 2 | 3 | | 0 | 3 | | 2 | | 1 | | | 2 | | 0 | | 2 | | |  | | | 2 | 3 | |  | | 0 | | 3 | | 25 | 60 |
| 7 | Knettel et al. (40) | | 1 | 2 | 3 | | 3 | 3 | | 2 | | 2 | | | 0 | | 3 | | 3 | | |  | | | 3 | 3 | |  | | 3 | | 3 | | 34 | 81 |
| 8 | Kubota et al. (75) | | 1 | 2 | 3 | | 0 | 3 | | 1 | | 3 | | | 0 | | 1 | | 2 | | |  | | | 3 | 3 | |  | | 0 | | 2 | | 24 | 57 |
| 9 | Gordon et al. (72) | | 1 | 3 | 3 | | 3 | 3 | | 2 | | 1 | | | 3 | | 3 | | 3 | | |  | | | 3 | 3 | |  | | 0 | | 3 | | 34 | 81 |
| 10 | Jones, Rodriguez, Alcaide, Weiss & Peltzer (121) | | 1 | 0 | 3 | | 0 | 3 | | 1 | | 2 | | | 0 | | 3 | | 0 | | |  | | | 3 | 3 | |  | | 0 | | 2 | | 21 | 50 |
| 11 | Takegata, Takeda, Sakanashi, Tanaka & Kitamura (70) | | 3 | 2 | 3 | | 0 | 3 | | 3 | | 2 | | | 2 | | 1 | | 3 | | |  | | | 2 | 2 | |  | | 0 | | 3 | | 29 | 69 |
| 12 | Giallo et al. (43) | | 0 | 3 | 3 | | 3 | 3 | | 3 | | 2 | | | 3 | | 0 | | 3 | | |  | | | 3 | 2 | |  | | 0 | | 3 | | 31 | 74 |
| 13 | Rodriguez et al. (49) | | 3 | 2 | 3 | | 0 | 3 | | 2 | | 3 | | | 1 | | 2 | | 2 | | |  | | | 3 | 3 | |  | | 1 | | 3 | | 31 | 74 |
| 14 | Shi, Ren, Li & Dai (61) | | 1 | 3 | 3 | | 0 | 3 | | 2 | | 2 | | | 2 | | 1 | | 2 | | |  | | | 3 | 1 | |  | | 0 | | 2 | | 25 | 60 |
| 15 | Bodnar-Deren, Klipstein, Fersh, Shemesh & Howell (80) | | 0 | 3 | 3 | | 1 | 3 | | 3 | | 2 | | | 1 | | 0 | | 3 | | |  | | | 3 | 3 | |  | | 2 | | 2 | | 29 | 69 |
| 16 | Muzik, Brier, Menke, Davis & Sexton (82) | | 3 | 3 | 2 | | 0 | 3 | | 1 | | 0 | | | 0 | | 0 | | 3 | | |  | | | 3 | 3 | |  | | 0 | | 3 | | 24 | 57 |
| 17 | Fisher et al. (50) | | 2 | 3 | 3 | | 1 | 3 | | 3 | | 1 | | | 2 | | 0 | | 3 | | |  | | | 3 | 3 | |  | | 0 | | 1 | | 28 | 67 |
| 18 | Crandall, Sridharan & Schermer(123) | | 0 | 0 | 2 | | 0 | 3 | | 1 | | 1 | | | 1 | | 0 | | 0 | | |  | | | 3 | 2 | |  | | 0 | | 3 | | 16 | 38 |
| 19 | Abdelghani et al. (124) | | 3 | 2 | 3 | | 2 | 3 | | 1 | | 0 | | | 1 | | 0 | | 3 | | |  | | | 3 | 3 | |  | | 0 | | 3 | | 27 | 64 |
| 20 | Faisal-Cury, Levy & Matijasevich (73) | | 3 | 3 | 3 | | 0 | 2 | | 3 | | 2 | | | 2 | | 3 | | 3 | | |  | | | 3 | 3 | |  | | 0 | | 3 | | 33 | 79 |
| 21 | Zewdu, Reta, Yigzaw & Tamirat (85) | | 2 | 2 | 2 | | 1 | 2 | | 2 | | 0 | | | 1 | | 0 | | 3 | | |  | | | 3 | 3 | |  | | 0 | | 1 | | 22 | 52 |
| 22 | Akram, Ahmed, Maqsood & Bibi (78) | | 2 | 3 | 3 | | 1 | 3 | | 2 | | 0 | | | 1 | | 3 | | 3 | | |  | | | 3 | 3 | |  | | 0 | | 1 | | 28 | 67 |
| 23 | Iyengar, Bondade & Raj (47) | | 2 | 2 | 2 | | 0 | 2 | | 2 | | 0 | | | 0 | | 0 | | 3 | | |  | | | 3 | 1 | |  | | 0 | | 1 | | 18 | 43 |
| 24 | Kalmbach et al.(92) | | 3 | 3 | 3 | | 0 | 3 | | 3 | | 1 | | | 2 | | 0 | | 2 | | |  | | | 3 | 3 | |  | | 0 | | 3 | | 29 | 69 |
| 25 | Zhang et al. (125) | | 3 | 3 | 3 | | 0 | 3 | | 1 | | 3 | | | 1 | | 3 | | 3 | | |  | | | 3 | 3 | |  | | 0 | | 2 | | 31 | 74 |
| 26 | Doi & Fujiwara (44) | | 1 | 2 | 1 | | 0 | 3 | | 3 | | 1 | | | 3 | | 0 | | 3 | | |  | | | 3 | 2 | |  | | 0 | | 2 | | 24 | 57 |
| 27 | Duan et al. (97) | | 1 | 3 | 3 | | 0 | 3 | | 0 | | 0 | | | 0 | | 3 | | 3 | | |  | | | 3 | 3 | |  | | 0 | | 2 | | 24 | 57 |
| 28 | Islam et al. (48) | | 3 | 3 | 3 | | 3 | 3 | | 3 | | 2 | | | 0 | | 3 | | 2 | | |  | | | 3 | 3 | |  | | 0 | | 3 | | 34 | 81 |
| 29 | Rurangirwa, Mogren, Ntaganira, Govender & Krantz (126) | | 1 | 3 | 3 | | 3 | 3 | | 3 | | 3 | | | 0 | | 1 | | 3 | | |  | | | 3 | 3 | |  | | 0 | | 2 | | 31 | 74 |
| 30 | Tabb et al. (84) | | 2 | 3 | 3 | | 0 | 3 | | 1 | | 2 | | | 2 | | 1 | | 3 | | |  | | | 3 | 2 | |  | | 0 | | 3 | | 28 | 67 |
| 31 | Rodriguez, Cook, Peltzer & Jones (122) | | 3 | 2 | 3 | | 0 | 3 | | 1 | | 2 | | | 2 | | 3 | | 2 | | |  | | | 3 | 1 | |  | | 1 | | 2 | | 28 | 67 |
| 32 | Castro e Couto et al. (81) | | 0 | 2 | 1 | | 0 | 2 | | 1 | | 0 | | | 0 | | 0 | | 3 | | |  | | | 3 | 1 | |  | | 0 | | 2 | | 15 | 36 |
| 33 | Shamu, Zarowsky, Roelens, Temmerman & Abrahams (127) | | 2 | 1 | 2 | | 0 | 3 | | 1 | | 1 | | | 1 | | 2 | | 3 | | |  | | | 3 | 2 | |  | | 1 | | 2 | | 24 | 57 |
| 34 | Zhong et al. (128) | | 1 | 3 | 3 | | 0 | 3 | | 1 | | 1 | | | 2 | | 1 | | 3 | | |  | | | 3 | 2 | |  | | 0 | | 3 | | 26 | 62 |
| 35 | Alhusen, Frohman & Purcell (129) | | 2 | 3 | 3 | | 0 | 3 | | 3 | | 2 | | | 1 | | 2 | | 2 | | |  | | | 3 | 3 | |  | | 0 | | 2 | | 29 | 69 |
| 36 | Fonseca-Machado, Alves, Haas, Monteiro & Gomes-Sponholz (130) | | 2 | 3 | 3 | | 3 | 3 | | 3 | | 2 | | | 0 | | 0 | | 3 | | |  | | | 3 | 2 | |  | | 0 | | 1 | | 28 | 67 |
| 37 | Peltzer (79) | | 1 | 2 | 3 | | 0 | 3 | | 2 | | 1 | | | 1 | | 0 | | 2 | | |  | | | 3 | 1 | |  | | 0 | | 1 | | 23 | 55 |
| 38 | Sit et al. (41) | | 2 | 3 | 3 | | 0 | 3 | | 3 | | 2 | | | 3 | | 0 | | 2 | | |  | | | 3 | 3 | |  | | 0 | | 2 | | 29 | 69 |
| 39 | Farias et al. (67) | | 0 | 2 | 3 | | 0 | 3 | | 2 | | 0 | | | 2 | | 0 | | 3 | | |  | | | 3 | 1 | |  | | 0 | | 2 | | 21 | 50 |
| 40 | Tavares et al. (60) | | 0 | 2 | 2 | | 0 | 3 | | 1 | | 0 | | | 1 | | 0 | | 3 | | |  | | | 3 | 3 | |  | | 0 | | 1 | | 19 | 45 |
| 41 | Benute et al. (91) | | 0 | 2 | 3 | | 0 | 3 | | 2 | | 0 | | | 0 | | 0 | | 1 | | |  | | | 2 | 1 | |  | | 0 | | 0 | | 14 | 33 |
| 42 | Gavin, Tabb, Melville, Guo & Katon (62) | | 0 | 2 | 2 | | 0 | 3 | | 3 | | 2 | | | 2 | | 0 | | 3 | | |  | | | 3 | 3 | |  | | 0 | | 3 | | 26 | 62 |
| 43 | Paris, Bolton & Weinberg (64) | | 3 | 3 | 2 | | 0 | 2 | | 3 | | 2 | | | 3 | | 0 | | 3 | | |  | | | 3 | 3 | |  | | 0 | | 3 | | 30 | 71 |
| **Suicidal and self-harm ideation and attempt** | | | | | | | | | | | | | | | | | | | | | | | | | | | | | | | | | | | |
| 44 | Maré et al. (39) | | 1 | 2 | 3 | | 0 | 3 | | 1 | | 2 | | | 0 | | 0 | | 3 | | |  | | | 3 | 3 | |  | | 0 | | 3 | | 24 | 57 |
| 45 | Szpunar, Crawford, Baca & Lang (66) | | 2 | 3 | 3 | | 0 | 3 | | 3 | | 1 | | | 3 | | 3 | | 3 | | |  | | | 3 | 2 | |  | | 0 | | 3 | | 32 | 76 |
| 46 | Martini et al. (46) | | 1 | 3 | 3 | | 0 | 3 | | 1 | | 1 | | | 3 | | 2 | | 3 | | |  | | | 3 | 3 | |  | | 0 | | 3 | | 29 | 69 |
| 47 | Belete, Kassew, Demilew & Zeleke (77) | | 1 | 2 | 3 | | 3 | 3 | | 3 | | 3 | | | 3 | | 1 | | 3 | | |  | | | 3 | 3 | |  | | 1 | | 2 | | 34 | 81 |
| 48 | Kugbey et al. (54) | | 2 | 2 | 3 | | 3 | 3 | | 2 | | 1 | | | 1 | | 1 | | 3 | | |  | | | 3 | 3 | |  | | 0 | | 2 | | 29 | 69 |
| 49 | Palfreyman (65) | | 2 | 2 | 3 | | 2 | 3 | | 3 | | 3 | | | 3 | | 1 | | 3 | | |  | | | 3 | 3 | |  | | 2 | | 3 | | 36 | 86 |
| 50 | Belete & Misgan (56) | | 2 | 2 | 3 | | 0 | 2 | | 1 | | 2 | | | 3 | | 3 | | 2 | | |  | | | 3 | 2 | |  | | 0 | | 2 | | 27 | 64 |
| 51 | Levey et al. (45) | | 0 | 2 | 3 | | 0 | 3 | | 3 | | 0 | | | 1 | | 0 | | 3 | | |  | | | 3 | 3 | |  | | 0 | | 2 | | 23 | 54 |
| 52 | Onah, Field, Bantjes & Honikman (83) | | 1 | 2 | 3 | | 2 | 3 | | 2 | | 2 | | | 3 | | 2 | | 3 | | |  | | | 3 | 3 | |  | | 0 | | 3 | | 32 | 76 |
| 53 | Supraja et al. (55) | | 0 | 2 | 3 | | 0 | 3 | | 1 | | 1 | | | 0 | | 0 | | 3 | | |  | | | 3 | 2 | |  | | 0 | | 3 | | 21 | 50 |
| 54 | Asad et al. (52) | | 1 | 3 | 3 | | 0 | 2 | | 2 | | 1 | | | 2 | | 1 | | 3 | | |  | | | 3 | 3 | |  | | 0 | | 2 | | 26 | 62 |
| 55 | Mezey, Bacchus, Bewley & White (53) | | 2 | 3 | 3 | | 3 | 3 | | 3 | | 1 | | | 2 | | 0 | | 3 | | |  | | | 3 | 3 | |  | | 0 | | 1 | | 30 | 71 |
| 56 | Farber, Herbert & Reviere (38) | | 2 | 3 | 2 | | 0 | 2 | | 2 | | 2 | | | 0 | | 0 | | 1 | | |  | | | 2 | 1 | |  | | 0 | | 3 | | 20 | 48 |
| **Suicide attempt** | | | | | | | | | | | | | | | | | | | | | | | | | | | | | | | | | | | |
| 57 | Gressier et al. (28) | | 1 | 2 | 3 | | 0 | 2 | | 2 | | 2 | | | 0 | | 0 | | 3 | | |  | | | 3 | 3 | |  | | 0 | | 3 | | 24 | 57 |
| **Suicide death** | | | | | | | | | | | | | | | | | | | | | | | | | | | | | | | | | | | |
| 58 | Adu, Brown, Asaolu & Sanderson (57) | | 0 | 2 | 3 | | 0 | 3 | | 3 | | 3 | | | 3 | | 0 | | 3 | | |  | | | 3 | 3 | |  | | 0 | | 3 | | 29 | 69 |
| 59 | Gold, Singh, Marcus & Palladino (58) | | 0 | 2 | 3 | | 0 | 3 | | 3 | | 3 | | | 3 | | 0 | | 3 | | |  | | | 3 | 3 | |  | | 0 | | 3 | | 29 | 69 |
| **% of studies rated as 1, 2 or 3** | | | 80 | 97 | 100 | | 29 | 100 | | 98 | | 75 | | | 73 | | 46 | | 97 | | |  | | | 100 | 100 | |  | | 14 | | 98 | |  |  |
|  | | |  | | | | | | | | | | | | | | | | | | | | | | | | | | | | | | | | |
|  | |  | ‘high’ quality (>75%) | | | 0 = not at all | | | | | | | | | | | | | | | | | | | | | | | | | | | | | |
|  | |  | ‘good’ quality (50-75%) | | | 1 = very sightly | | | | | | | | | | | | | | | | | | | | | | | | | | | | | |
|  | |  | ‘moderate’ quality (25-49%) | | | 2 = moderately | | | | | | | | | | | | | | | | | | | | | | | | | | | | | |
|  | |  | ‘poor’ quality (<25%) | | | 3 = complete | | | | | | | | | | | | | | | | | | | | | | | | | | | | | |
|  |  | |  | | | | | |  | |  | |  |  | |  | |  | |  |  | |  |  | |  |  | |  | |  | |  |  |  |
